# Analysis of a Novel Bacteriophage vB_AchrS_AchV4 Highlights the Diversity of *Achromobacter* Viruses

**DOI:** 10.3390/v13030374

**Published:** 2021-02-27

**Authors:** Laura Kaliniene, Algirdas Noreika, Algirdas Kaupinis, Mindaugas Valius, Edvinas Jurgelaitis, Justas Lazutka, Rita Meškienė, Rolandas Meškys

**Affiliations:** 1 Department of Molecular Microbiology and Biotechnology, Institute of Biochemistry, Life Sciences Center, Vilnius University, Saulėtekio av. 7, LT-10257 Vilnius, Lithuania; algirdas.noreika@gmc.vu.lt (A.N.); edvinas.jurgelaitis@chgf.stud.vu.lt (E.J.); rita.meskiene@bchi.vu.lt (R.M.); rolandas.meskys@bchi.vu.lt (R.M.); 2 Proteomics Centre, Institute of Biochemistry, Life Sciences Centre, Vilnius University, Saulėtekio av. 7, LT-10257 Vilnius, Lithuania; algirdas.kaupinis@gf.vu.lt (A.K.); mindaugas.valius@bchi.vu.lt (M.V.); 3 Department of Eukaryote Gene Engineering, Institute of Biotechnology, Life Sciences Center, Vilnius University, Saulėtekio av. 7, LT-10257 Vilnius, Lithuania; justas.lazutka@bti.vu.lt

**Keywords:** bacteriophage, *Achromobacter*, *Siphoviridae*

## Abstract

*Achromobacter* spp. are ubiquitous in nature and are increasingly being recognized as emerging nosocomial pathogens. Nevertheless, to date, only 30 complete genome sequences of *Achromobacter* phages are available in GenBank, and nearly all of those phages were isolated on *Achromobacter xylosoxidans*. Here, we report the isolation and characterization of bacteriophage vB_AchrS_AchV4. To the best of our knowledge, vB_AchrS_AchV4 is the first virus isolated from *Achromobacter spanius*. Both vB_AchrS_AchV4 and its host, *Achromobacter spanius* RL_4, were isolated in Lithuania. VB_AchrS_AchV4 is a siphovirus, since it has an isometric head (64 ± 3.2 nm in diameter) and a non-contractile flexible tail (232 ± 5.4). The genome of vB_AchrS_AchV4 is a linear dsDNA molecule of 59,489 bp with a G+C content of 62.8%. It contains no tRNA genes, yet it includes 82 protein-coding genes, of which 27 have no homologues in phages. Using bioinformatics approaches, 36 vB_AchrS_AchV4 genes were given a putative function. A further four were annotated based on the results of LC–MS/MS. Comparative analyses revealed that vB_AchrS_AchV4 is a singleton siphovirus with no close relatives among known tailed phages. In summary, this work not only describes a novel and unique phage, but also advances our knowledge of genetic diversity and evolution of *Achromobacter* bacteriophages.

## 1. Introduction

*Achromobacter* spp. are ubiquitous, lactose nonfermenting, Gram-negative bacilli that are frequently isolated from a wide range of environmental habitats [1]. The intrinsic characteristics of Achromobacteria, such as the large genome rich in C-G sequences, resistance to arsenic and other toxic metals, and the ability to degrade aromatic compounds, enable survival in adverse environmental conditions that would otherwise limit distribution [2,3,4]. Although *Achromobacter* species do not typically cause diseases in normal subjects, some members of this genus may become opportunistic pathogens in certain conditions, such as cystic fibrosis, hematologic and solid organ malignancies, renal failure, and certain immune deficiencies. In such cases, infections caused by this bacterium are complicated by the fact that *Achromobacter* spp. possess innate antimicrobial resistance, readily acquire adaptive resistance with antimicrobial exposure, and tend to alter the expression of certain genes to promote chronic infection [2,3,4,5].

The genus *Achromobacter*, described by Yabuuchi and Yano in 1981 [6], originally contained a single species, *Achromobacter xylosoxidans*. To date, a total of 20 *Achromobacter* species and 24 genogroups belong to this genus [7,8]; however, knowledge on bacteriophages targeting this bacterium is still scarce. While a number of published reports of *Achromobacter* phages may be found in the literature [9,10,11,12,13,14,15,16,17], nearly all describe viruses that are lytic against *Achromobacter xylosoxidans*, the type species of the genus. To date, there have been no reports of bacteriophages active on *Achromobacter spanius*. *A. spanius* have been identified in various samples, including those from freshwater and soil, and in human clinical isolates. The type strain, *A. spanius* LMG 5911, originated from the blood of a cystic fibrosis patient [18,19].

In this work, we describe a novel virus vB_AchrS_AchV4 isolated on *A. spanius* RL_4. Comparative genomic analyses reveal that bacteriophage vB_AchrS_AchV4 (subsequently referred to by its shorter common laboratory name, AchV4) is a singleton siphovirus, which is distinct from all other known bacterial viruses. The results presented here highlight not only the uniqueness of AchV4 but also the diversity of *Achromobacter* phages sequenced to date.

## 2. Materials and Methods

### 2.1. Phage Techniques

Both bacteriophage AchV4 and its host, *Achromobacter spanius* isolate RL_4 (verified by 16S rRNA sequencing, NCBI accession number, MT670403), were isolated from the outwash of grapes (*Vitis vinifera*) harvested from a private garden (54°47’23.28’’ N/25°22’59.196’’ E; Vilnius, Lithuania). A standard sample enrichment technique was used [20], with incubation at 22 °C. For phage purification, bacteria were cultivated aerobically in LB at 30 °C, and the phage was purified from a single plaque isolate using the soft-agar overlay method as described in [21]. The purification process was repeated three consecutive times. Then, phage suspension was subjected to CsCl gradient (densities: 1.1 g/mL^−1^, 0.9 g mL^−1^, 0.7 g mL^−1^, 0.5 g mL^−1^) centrifugation at 24,000 rpm for 3 h (11 °C) using Spinco SW39 rotor (Beckman Instruments, Inc., Fullerton, CA, USA) as described in [21]. For host range determination, the bacterial strains used in this study (listed in Table S1, described in [22]) were cultivated aerobically in LB at 30 °C, with the exception of *Burkholderia sp.* MAK1 [23]. The latter bacterium was cultivated in liquid EFA medium (10.0 g L^−1^ K_2_HPO_4_, 4.0 g L^−1^ KH2PO4, 0.5 g L^−1^ yeast extract, 1.0 g L^−1^ (NH_4_)2SO_4_, 0.2 g L^−1^ MgSO_4_·7H_2_O (pH 7.0)) supplemented with salt solution (2.0 g L^−1^ CaCl_2_·2H_2_O, 1.0 g L^−1^ MnSO_4_·4H_2_O, 0.5 g L^−1^ FeSO_4_·7H_2_O; all components were dissolved in 0.1 M HCl) and with an appropriate carbon source (1.0 g L^−1^ succinate).

To determine the burst size and latency period of AchV4, a one-step growth curve was carried out as described in [15]. To determine the nature of the AchV4 receptor, *Achromobacter spanius* RL_4 cells were treated with sodium acetate (50 mM, pH 5.2) containing periodate (100 mM IO^4−^) at room temperature for 2 h or proteinase K (0.2 mg mL^−1^; Promega, Madison, WI, USA) at 37  °C for 3 h. The phage adsorption assay was then performed as described in [24]. Briefly, treated cells were harvested by centrifugation, washed three times with LB (2 ml), centrifuged again, and suspended in liquid LB medium so as to obtain an A_600_ of 0.7. The cells were then incubated with phage AchV4 (30 °C, 0.5 h). Afterwards, the cell-phage suspension was centrifuged to separate the unabsorbed phage particles (supernatant) from the cell/adsorbed phage fraction (sediment). The supernatant was then titrated.

### 2.2. TEM Analysis

The morphology of CsCl-purified phage particles was examined using a MorgagniTM 268(D) transmission electron microscope (FEI, Hillsboro, OR, USA), as described previously [21].

### 2.3. DNA Isolation

For the isolation of phage DNA, aliquots of phage suspension (10^11^–10^12^ PFU mL^−1^) were subjected to phenol/chloroform extraction and ethanol precipitation as described by Carlson and Miller [25]. Isolated phage DNA was subsequently subjected to genome sequencing and PCR.

### 2.4. Genome Sequencing and Assembly

The isolated genomic DNA of AchV4 (~100 μg) was sent to BaseClear (Leiden, Netherlands) for genome sequencing, quality assessment of the sequence reads, and de novo assembly. There, the single-end reads were generated using the Illumina NovaSeq 6000 (San Diego, CA, USA) system. FASTQ read sequence files were generated using bcl2fastq2 version 2.18 (Illumina). Initial quality assessment was based on data passing the Illumina Chastity filtering. Subsequently, reads containing PhiX control signal were removed using an in-house filtering protocol. In addition, reads containing (partial) adapters were clipped (up to a minimum read length of 50 bp). The second quality assessment was based on the remaining reads using the FASTQC quality control tool version 0.11.5. SPAdes v3.10 [26] was used to assemble the error corrected reads into contigs, which were linked together and placed into scaffolds using SSPACE v2.3 [27]. Gap-closure and assembly polishing was performed using GapFiller v1.10 [28] and Pilon v1.21 [29].

Thus, the genome of AchV4 was assembled de novo into a single viral contig with an average coverage of 163.81 that was visualized and analyzed using Geneious Prime v2021.01 (https://www.geneious.com, accessed on 15 January 2021). PCR off the contig ends (forward primer, 5’- CCCTCAAGACACTTCAGCAGA, and reverse primer, 5’-GCGAAAACCTATCGTGTC), followed by Sanger sequencing, was used to close and confirm the genome sequence. Since no defined genomic termini were identified by PhageTerm (v1.0.12, Galaxy, https://galaxy.pasteur.fr/, accessed on 15 January 2021) and the genome produced a circular assembly, it was reopened at a locus close to the small terminase gene, as recommended previously [30]. The complete genome sequence of *Achromobacter* phage AchV4 was deposited in GenBank under accession number MW269554. The raw reads may be found at the NCBI Sequence Read Archive (SRA), accession number SRX8646550.

### 2.5. Genome Sequence Analysis

The protein-coding genes in the genome of AchV4 were predicted using Glimmer3 (Geneious Prime v2021.01), and then reviewed manually. Analysis of the genome sequence was performed using BLAST, PSI-BLAST, and Megablast (https://blast.ncbi.nlm.nih.gov/Blast.cgi, accessed on 15 January 2021), ProDom, TIGRFAM, SignalP, TMHMM v2.0, LipoP v1.0, Galaxy [31], as well as HHPred, HHblits, and HHsenser [32]. To search for tRNAs, tRNAscan-SE 1.21 [33] was used, whereas PhagePromoter (Galaxy), ARNold (http://rssf.i2bc.paris-saclay.fr/toolbox/arnold/, accessed on 15 January 2021), and PHACTS [34] were used to predict promoters, rho-independent terminators and the lifestyle of the phage, respectively. Spanin-coding genes were searched for by following the method described in [35].

### 2.6. Phylogeny

Phylogenetic and molecular evolutionary analyses were conducted using MEGAX version 10.1.8 [36], VIRFAM [37], ViPTree [38], SplitsTree4 (v4.16.2), VICTOR [39], and ANI (Average Nucleotide Identity)/AAI-(Average Amino Acid Identity) Matrix calculator (http://enve-omics.ce.gatech.edu, accessed on 13 December 2020). The CoreGenes5.0 webserver (https://coregenes.ngrok.io/, accessed on 10 January 2021) was used (with default parameters) to calculate the number of shared protein-coding genes.

### 2.7. Proteomic Analysis

Analysis of AchV4 virion structural proteins was performed following a modified filter-aided sample preparation (FASP) protocol as described in [40].

## 3. Results

### 3.1. Phage Isolation and Characterization

**Figure 1:**
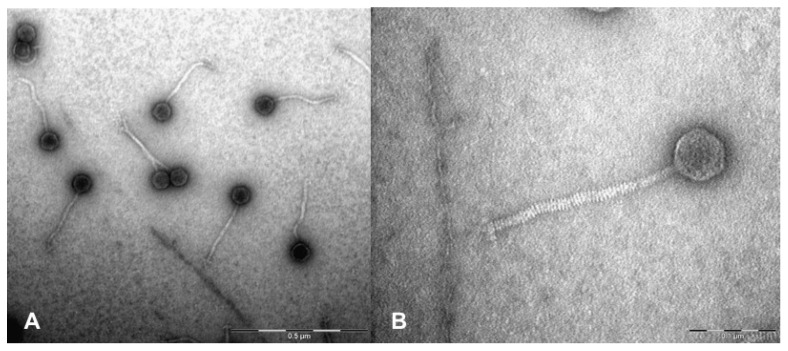
Electron micrographs of negatively stained AchV4 particles. (**A**) TEM micrograph showing CsCl-purified AchV4 virions (the scale bar corresponds to 0.5 μm); (**B**) a close-up view of an AchV4 particle (the scale bar corresponds to 0.1 μm).

Both bacteriophage AchV4 and its host, *Achromobacter spanius* isolate RL_4 were isolated in Lithuania, as described in Materials and Methods. Electron microscopy revealed (Figure 1) that AchV4 likely belongs to the family *Siphoviridae* since it is characterized by an isometric head (64 ± 3.2 nm in diameter; *n* = 10) and a non-contractile flexible tail (232 ± 5.4 nm in length, 15 ± 0.5 nm in width; *n* = 10).

On a lawn of RL_4, the phage AchV4 formed clear plaques, with a diameter of about 1.5 mm (Figure S1). Based on a one-step growth curve analysis, AchV4 has a latent period of approximately 80 minutes and an average burst size of 100 phage particles per infected cell (Figure S2).

In total, 17 strains of Beta- and Gammaproteobacteria were used to explore the host-range of AchV4. As seen in Table S1, only the cells of *Achromobacter spanius* RL_4 were susceptible to infection by AchV4, suggesting that this phage probably has a narrow host range.

The infection process begins with the adsorption of the phage particle to the bacterial surface. During adsorption, phages target a wide range of host receptors, including proteins, sugars and even certain cell surface structures, such as flagella, pili and capsules [41]. To determine the nature of the receptor used by AchV4, *A. spanius* RL_4 cells were treated with either periodate (which destroys carbohydrate but leaves protein unaltered) or proteinase K (destroys protein but leaves LPS unaltered) prior to phage adsorption assay. As seen in Figure S3, when AchV4 was preincubated with proteinase K-treated cells, only a small fraction of unabsorbed phage particles remained in the supernatant after centrifugation, suggesting that the AchV4 receptor is not a protein. In contrast, in the case of periodate-treated RL_4 cells, a large number of free phage particles found in the supernatant after centrifugation indicated that AchV4 was incapable of binding to periodate-treated cells. Taken together, the results of the adsorption assay suggest that AchV4 uses LPS as a receptor to bind to *A. spanius* RL_4 cells.

### 3.2. AchV4 Genome

**Figure 2:**
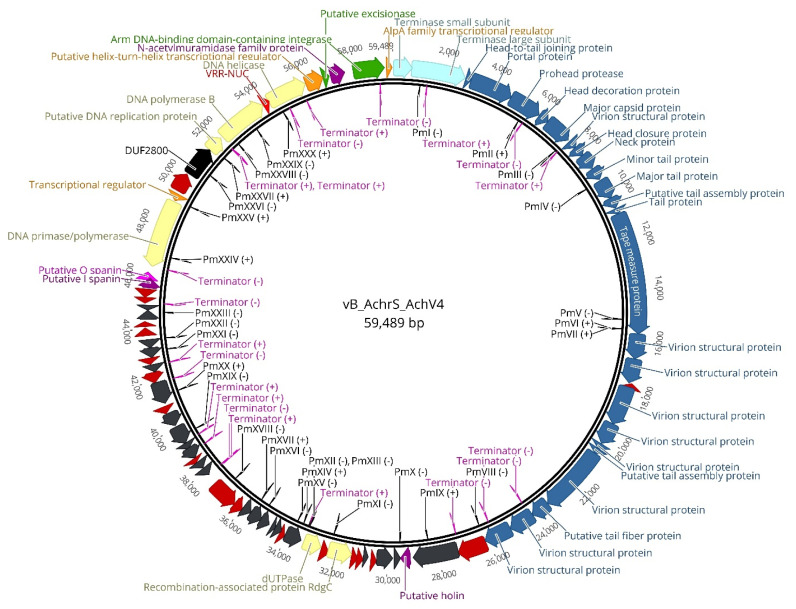
Functional genome map of bacteriophage AchV4. The coding capacity of the AchV4 genome is shown. Functions were assigned based on homology to known genes and/or MS-MS analysis. The color code is as follows: blue, structural proteins and those involved in virion morphogenesis; light blue, terminase complex; yellow, DNA metabolism; orange, transcription; violet/purple, lysis; green, lysogeny; red, genes that encode unique proteins with no reliable homology to database entries; grey, genes of unknown function. Pm—promoter. The figure was generated using Geneious Prime 2021.01.

Phage AchV4 has a linear dsDNA genome consisting of 59,489 bp with a G+C content of 62.8%, which is similar to 64.9% reported for *Achromobacter spanius* [18]. The results of PCR and PhageTerm analysis indicate that the genome is circularly permuted (data not shown). MegaBLAST analysis of the complete genome sequence of AchV4 against viral DNA (Viruses, taxid:10239) returned no hits, suggesting that this phage represents a new species that is not closely related to any recognized phage groups. A total of 82 protein-coding genes (ranging from 177 to 4764 bp), 30 promoters, and 20 Rho-independent terminators were predicted for AchV4 (Figure 2, Table S2–S4), yet no tRNAs were identified, indicating that the phage completely relies on the host tRNA for its protein synthesis.

On the basis of BLASTP, HHpred, and conserved domain analysis, only 36 of the AchV4 genes could be assigned a putative function. The results of BLASTP analysis revealed that 18 AchV4 genes (22% of AchV4 coding capacity) code for unique proteins that have no reliable identity to database entries (E values of > 0.0001), whereas as many as 27 (33%) have no homologues in phages. Moreover, out of 56 AchV4 gene products that did have homologues in viruses, 34 were most closely related to bacterial rather than phage-encoded proteins (Table S2). In addition, because the genome of AchV4 diverges from all other available phage genomes, the range of similarity shared by AchV4-encoded proteins and those found in other phages was, in most cases (41 out of 56 predicted gene products), as low as 24–50% at the amino acid level. The highest identity values were found for: the head-to-tail joining protein gpAchV4_0003 (61% identity with the corresponding protein from *Synechococcus* virus S-ESS1), the neck protein gpAchV4_0010 (61% with the hypothetical protein AH2_00059 from *Burkholderia* phage vB_BceS_AH2), the major tail protein gpAchV4_0007 (61% with the hypothetical protein AH2_00057 from *Burkholderia* phage vB_BceS_AH2), the putative tail protein gpAchV4_00014 (61% with the corresponding protein from *Burkholderia* phage vB_BceS_AH2), two hypothetical proteins encoded by AchV4_0042 (62% with the hypothetical protein HOS31_gp44 found in *Bordetella* phage FP1) and AchV4_0060 (62% with the hypothetical protein HOV23_gp100 of *Pseudomonas* phage Lana), and the putative excisionase encoded by AchV4_0079 (76% identity with that of *Klebsiella* phage YMC16/01/N133_KPN_BP).

#### 3.2.1. Structural Module

As observed with many tailed phages, the AchV4 genome displays a modular organization, with genes of related function clustered together (Figure 2). The structural module, which occupies nearly half of the AchV4 genome, encompasses 25 genes that encode structural proteins and those involved in virion assembly as well as one hypothetical protein, unique to AchV4. The architecture of this module in AchV4 shows features that are typical of most siphoviruses: the head and tail genes are found on the same DNA strand, with the head genes located just upstream of the tail genes. Nevertheless, according to Virfam, the head-neck-tail module of AchV4 belongs to *Siphoviridae* of Type 1, Cluster 6, which is mostly composed of Proteobacteria-infecting viruses that exhibit a different head-neck-tail gene order compared to that observed in the majority of siphoviruses. Indeed, as seen in Figure 2, the gene coding for AchV4 Ad1 (head-to-tail joining protein) is found between those coding for the terminase and portal proteins, whereas in most Type 1 siphoviruses, *ad*1 is usually located downstream from the major capsid protein gene.

Based on the results of bioinformatics and proteomic analyses, the AchV4 virus particle is built with at least 21 structural proteins. As seen in Table S2, six AchV4 genes were predicted to be responsible for capsid formation, including those coding for the head-to-tail joining protein (AchV4_0003), portal protein (AchV4_0004), prohead protease (AchV4_0005), head decorator protein (AchV4_0006) as well as the major capsid (AchV4_0007) and head closure (AchV4_0009) proteins. The gene product of AchV4_0009 is a 133-aa protein that, based on BLASTP analysis, shows no similarity to phage structural proteins. Nevertheless, HHpred analysis revealed that gpAchV4_0009 may be related to the tail attachment protein gpFII of bacteriophage λ (HHPred probability, 99.3%; E value, 1.9 × 10^−11^), and the protein was also detected by LC–MS/MS (Table 1).

The gene immediately downstream from AchV4_0007 encodes a 115-aa protein that has no homologues in databases. However, based on its position on the genome and the fact that it has been identified as the virion structural protein by LC–MS/MS, the gene product of AchV4_0007 is likely a constituent of AchV4 head as well.

AchV4_00011–0026 make up the tail morphogenesis cluster, containing the genes encoding the major and minor tail proteins (AchV4_00012 and AchV4_00011, respectively), the tape measure protein (AchV4_00015), a putative tail fiber protein (AchV4_00024), eight virion structural proteins (AchV4_00016, AchV4_00017, AchV4_00019-AchV4_00023, AchV4_00025, and AchV4_00026) as well as two tail assembly proteins, namely gpAchV4_0013 and gpAchV4_0022. With the exception of gpAchV4_00011 and gpAchV4_00014, which were assigned as structural proteins by virtue of their percent identity to homologous sequences, all tail-forming proteins were detected by LC–MS/MS. Based on HHpred analysis, the minor tail protein encoded by AchV4_00011, which shares 46% amino acid sequence identity (E value, 7.98 × 10^−42^) with that of *Klebsiella* phage YMC16/01/N133_KPN_BP, may be related to the minor tail protein U from Enterobacteria phage λ (HHPred probability, 99.66%; E value, 1.4 × 10^−15^). The gene product of AchV4_00014, as discussed above, is one of seven AchV4 proteins with the highest similarity to phage proteins. Thus, the reason why gpAchV4_00011 and gpAchV4_00014 were not detected by LC–MS/MS is probably due to their low abundance in virions or to the incompatibility of these proteins with sample preparation procedures. Notably, LC–MS/MS identified two AchV4 structural proteins, namely, gpAchV4_00025 and gpAchV4_00026 (both encoded by the tail module genes), that show no similarity to characterized phage structural proteins in the NCBI database.

#### 3.2.2. DNA Packaging

Upstream from the head module lies the AchV4 DNA packaging module. In tailed phages, the packaging machine usually consists of a portal ring and a terminase complex. Most characterized terminases are heteroligomers that consist of a small subunit (TerS) involved in DNA recognition and a large terminase subunit (TerL) containing the ATPase and the endonuclease activities [42,43]. The DNA packaging motor, terminase of AchV4, is encoded by AchV4_0001 (TerS) and AchV4_0002 (TerL) that show similarity to the corresponding proteins of bacteriophage Phobos (46 and 60% aa identity, respectively). The gene product of AchV4_0004 is the Phage-Portal-2 superfamily (pfam05136) protein that shares 57% amino acid sequence identity with the portal protein of *Ruegeria* phage DSS3-P1 (E value, 0) and has also been identified by LC–MS/MS.

#### 3.2.3. Lysis Genes

In the case of dsDNA phages of Gram-negative hosts, the main players for cell lysis are holins, endolysins, and spanins [44,45]. The gene for a holing of AchV4 (AchV4_0029) was found downstream from the structural module. Based on the BLASTP analysis, a 134-aa Phage_holin_3_1 family (pfam05106) protein gpAchV4_0029, which contains three transmembrane regions (as predicted by PHOBIUS), shows similarity to a putative holin of *Aeromonas* myovirus phiO18P (42% aa identity; E value, 4.61 × 10^−15^). In tailed phages, the lysis genes are usually found in close proximity to each other and form a lysis cassette [44,45]. However, immediately upstream from AchV4_0029, a 579-aa hypothetical protein-encoding gene AchV4_0028 is located. The predicted protein of AchV4_0028, which has only 15 homologues in the NCBI database, shows similarity to the hypothetical protein pEpSNUABM08_36 of *Erwinia* phage pEp_SNUABM_08 (24% aa identity; E-value, 6.34 × 10^−8^) and contains no identifiable conserved domains, thus making AchV4_0028 an unlikely candidate for the endolysin gene. However, 28 kb downstream from AchV4_0028, the gene AchV4_0080 is found. The predicted Muramidase (pfam11860) superfamily protein (194 aa) encoded by AchV4_0080 shows similarity (50% aa identity; E value, 4 × 10^−59^) to the functionally characterized endolysin Gp110 from *Salmonella* phage 10 [46]. Gp110 has a modular structure with an enzymatically active domain and a short (65 aa) cell-wall-binding domain at the N-terminus, and has been shown to possess N-acetylmuramidase (lysozyme) activity cleaving the β-(1,4) glycosidic bond between N-acetylmuramic acid and N-acetylglucosamine residues. GpAchV4_0080 is by 70 aa shorter than Gp110 and, as a result, lacks the N-terminal cell-wall-binding domain usually observed in bacterial autolysins or phage endolysins [45]. However, the enzymatic activity of unique autolysin, Rv3717 of *Mycobacterium tuberculosis*, which also lacks a cell-wall-binding domain and instead utilizes a net positive charge in substrate binding, has been confirmed recently [47]. Thus, there is a strong possibility that the gene product of AchV4_0080 does indeed function as an endolysin. Notably, a similar organization of the lysis genes was observed in *Arthrobacter* bacteriophages Circum, Mudcat, CapnMurica, and Gordon, where the endolysin genes were also found located upstream of the terminase large subunit genes [48].

For many years, it was thought that the holin-endolysin system was sufficient for host lysis. However, it has been shown recently that a third functional class of lysis proteins, the spanins, are required to disrupt the outer membrane during the final step of Gram-negative host lysis [49]. Phages employ either two-component spanins or unimolecular spanins. Two-component spanins, such as Rz-Rz1 from phage λ, consist of an i-spanin (integral inner membrane protein) and an o-spanin (outer membrane lipoprotein). Both proteins interact via their C-termini to form complexes that reach from the inner membrane to the outer membrane. All characterized i-spanins have an N-terminal transmembrane domain, whereas the o-spanins have an N-terminal SPII signal and an N-terminal lipobox [35]. In contrast, unimolecular spanins, such as gp11 from phage T1, have an N-terminal lipoylation signal sequence and a C-terminal transmembrane domain to account for the topology requirements [35]. Protein secondary structure prediction and LipoP v1.0 analysis [31] allowed for the identification of the two-component spanin system in AchV4. The putative i-spanin is encoded by AchV4_0068 and has an N-terminal transmembrane domain, whereas gpAchV4_0069 codes for a putative lipoprotein that has an N-terminal SPII signal and an N-terminal lipobox [LAS]C. The spanin genes partially overlap, as is commonly found in the phages of Gram-negative hosts [35]. Rather uncommon, however, is the location of AchV4 spanin cluster. As seen in Figure 2, the two-component spanin system-coding genes are located just upstream from the primase-polymerase gene, at a distance of about 16 kb from the holin gene.

#### 3.2.4. DNA Metabolism

Bioinformatics approaches allowed for the identification of seven genes associated with DNA metabolism, suggesting that the genome of AchV4 is a semiautonomous replicon, which likely “borrows” a number of essential replisome proteins from the host. As seen in Figure 2, AchV4 DNA metabolism-associated genes are localized in two clusters separated by ~14 kb. The smaller of the two clusters contains three genes, AchV4_0036, AchV4_0037 and AchV4_0038. Recombination-associated protein RdgC encoded by AchV4_0036 shares 34% amino acid sequence identity (E value, 8.07 × 10^−55^) with the DNA recombination-dependent growth factor C of *Acidovorax* myovirus ACP17 and is related to RdgC from *Escherichia coli* (HHPred probability, 100%; E value, 1.4 × 10^−61^). In *E. coli*, RdgC has been shown to act as an inhibitor of RecA-mediated homologous recombination [50]. Gp AchV4_0037 is a 108 aa protein that has no conserved domains and no reliable homologues in the databases, whereas gpAchV4_0038, whose homologues are only found in two metagenomes of phage origin, is an NTP-PPase_dUTPase family (cl16941) protein related to dUTPase form *Campylobacter jejuni* (HHPred probability, 100%; E value, 3.5 × 10^−40^).

DNA primase/polymerase (AchV4_0070), a putative DNA replication protein (AchV4_0074), DNA polymerase B (AchV4_0075), a VRR_NUC superfamily protein (AchV4_0076), and SNF2/RAD54 helicase family protein (AchV4_0077) coding genes are co-localized in the larger of the two clusters. As commonly found in dsDNA phages, AchV4 codes for the archaeo-eukaryotic DNA primase/polymerase fused with a replicative SF3 helicase at the C-terminus (gpAchV4_0070). The product of AchV4_0074 is a DUF2815 family protein that shares 50% amino acid sequence identity (E value, 2.76 × 10^−49^) with the hypothetical protein of *Pseudomonas syringae* phage Phobos, and is homologous to a DUF2815 family protein (gpORF6) from Enterobacter phage Enc34 (HHPred probability, 100%; E value, 3.6 × 10^−34^). Since it has been shown recently [51] that the latter is a single-stranded DNA-binding protein (SSB), gpAchV4_0074 is likely an SSB protein as well. Notably, a DUF2800 family protein encoded by AchV4_0073, which based on BlastP analysis shows no similarity to known DNA replication/recombination proteins, has been predicted by Virfam to belong to the Rad52 family. This family includes the DNA single-strand annealing proteins related to Rad52, such as RecT, Red-beta and ERF, that function in RecA-dependent as well as RecA-independent DNA recombination pathways [52].

#### 3.2.5. Lysogeny Module

Throughout all phage experiments, AchV4 behaved like a typical lytic phage: (i) the phage produced clear, discreet plaques on the lawn of *Achromobacter spanius* RL_4; (ii) there was no decrease/inconsistencies in PFU during phage titration, even after four rounds of plaque purification during phage isolation; (iii) the phage propagated well both in liquid culture and in the traditional agar gels; and finally (iv) no stable lysogens could be recovered from RL_4. Nevertheless, bacteriophage AchV4 was non-confidently predicted by the PHACTS algorithm [34] as having a temperate lifestyle (averaged probability, 0.515 ± 0.04). Moreover, on the genome of AchV4, immediately downstream from the large DNA metabolism cluster, the lysogeny module was identified. Lysogeny generally proceeds through three steps: the establishment, maintenance and, potentially, induction of the lytic development [53]. Most molecular knowledge of lysogeny has been derived from a limited number of bacterial viruses, such as *E. coli* phages λ or Mu. In the case of lambdoid phages, two central components provide commitment to the outcome of the “lysis versus lysogeny” decision: λ repressor cI for lysogeny and a bistable genetic switch Cro for lytic growth [53,54]. The decision itself is determined by the transcriptional activator cII, which activates the transcription of both cI and integrase, and lysogeny is established. In the absence of cII, activation does not occur, and lytic growth proceeds [55]. For the excision of the integrated genome, another phage-encoded protein Xis is required [55]. At a glance, the lysogeny module of AchV4 contains at least three out of five genes that are considered hallmark lysogeny genes in λ: *c*I, *int*, and *xis*. The predicted HTH_XRE (Helix-turn-helix XRE) superfamily (cl22854) protein encoded by AchV4_0078 shares 58% amino acid sequence identity (E value, 2.43 × 10^−96^) with a putative HTH-domain-containing protein of *Klebsiella* phage YMC16/01/N133_KPN_BP, and, based on HHpred analysis, may be related to the λ repressor cI (probability, 98.1%; E value, 1.8 × 10^−5^). Another AchV4 protein, the product of AchV4_0071, may also be related to cI according to HHpred ((probability, 99%; E value, 9 × 10^−8^). Based on the results of BLASTP, gpAchV4_0071 has a C-terminal YdaS_antitoxin domain (which, again, is a member of conserved protein domain superfamily HTH_XRE), has homologues only in four phages, and shares 44% aa sequence identity with the hypothetical protein KPNN133_069 of *Klebsiella* phage YMC16/01/N133_KPN_BP. The integrase of AchV4 (gpAchV4_0081), which has an N-terminal Arm-DNA-bind_3 domain (pfam13356) and a C-terminal INT_P4_C domain cd00801, is an orthologue of phage λ integrase (HHPred probability, 100%; E value, 1.2 × 10^−33^) and shows similarity to the corresponding protein from *Pseudomonas* phage phiAH14b (43% aa identity, E value, 5 × 10^−107^). Meanwhile, the product of AchV4_0079 (whose homologues had only been found in three phages) was assigned as a putative excisionase solely by virtue of high percent identity to the corresponding protein of *Klebsiella* phage YMC16/01/N133_KPN_BP. GpAchV4_0079 is a small 77 aa protein, which has been predicted to contain no conserved domains, is more related to the regulatory protein Cox of *E. coli* phage P2 (HHPred probability, 99.1%; E value, 3.4 × 10^−9^) than it is to excisionase from λ (HHPred probability, 98.5%; E value, 2 × 10^−6^). However, the Cox protein from bacteriophage P2 is a small multifunctional DNA-binding protein that is involved in site-specific recombination leading to P2 prophage excision and functions as a transcriptional repressor of the P2 Pc promoter. Hence, P2 Cox is functionally equivalent to both Cro protein and exisionase of phage λ [56]. Notably, another AchV4 protein, the Phage_AlpA family (PF05930) protein encoded by AchV4_0082 that shares 43% aa identity with the transcriptional regulator Vis of Enterobacteria phage P4 (E value, 2.72 × 10^−11^), may also be related to P2 Cox (HHPred probability, 98.8%; E value, 4.6 × 10^−8^). To summarize, based on the results of bioinformatics analysis, *Achromobacter* phage AchV4, while clearly showing lytic activity toward *Achromobacter spanius* isolate RL_4 under standard laboratory conditions, is likely capable of lysogeny under different conditions and/or in the case of a different host.

### 3.3. Phylogenetic Analysis

**Figure 3:**
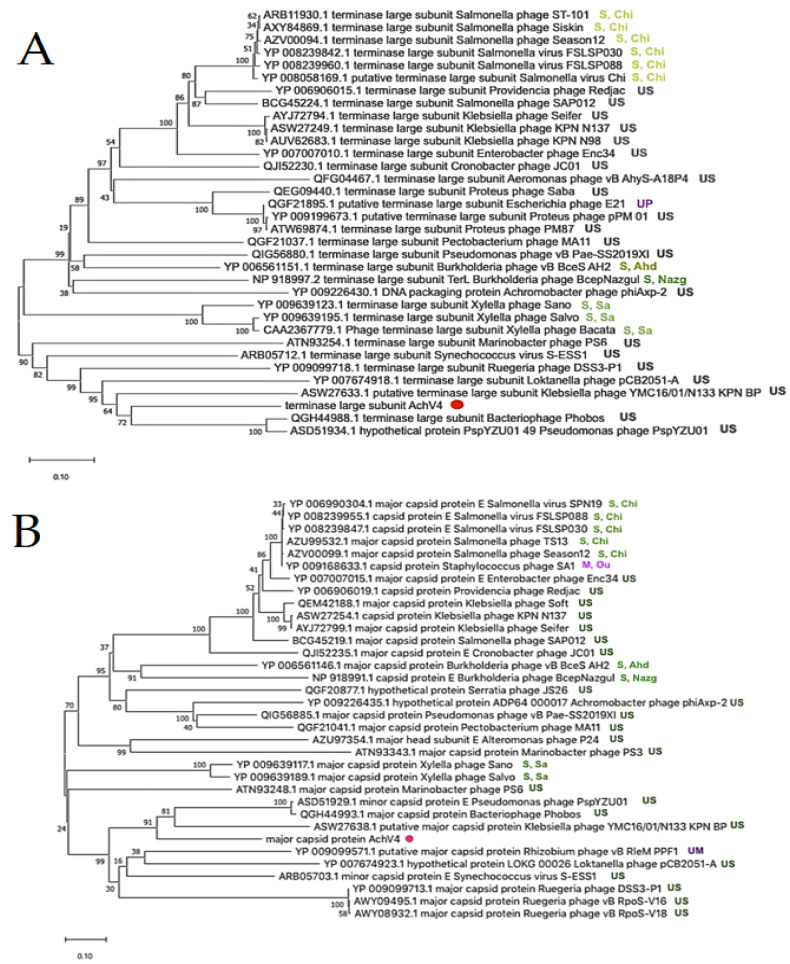
Phylogenetic analysis. Neighbor-joining tree analysis based on the ClustalW alignment of: the large terminase subunit (**A**) and the major capsid protein (**B**) sequences. The percentage of replicate trees in which the associated taxa clustered together in the bootstrap test (500 replicates) is shown next to the branches. Evolutionary analyses were conducted in MEGA X. US—unclassified siphovirus; UM—unclassified myovirus; UP—unclassified podovirus; S, Sa—*Siphoviridae*, *Sanovirus*; S, Chi—*Siphoviridae*, *Chivirus*; S, Ahd—*Siphoviridae*, *Ahduovirus*; S, Nazg—*Siphoviridae*; *Nazgulvirus*; M, Ou—*Myoviridae*; *Ounavirinae*; AchV4 is indicated by a red dot.

To explore the evolutionary relationships between AchV4 and other tailed phages, phylogenetic trees were generated for the major capsid protein (MCP) and the terminase large subunit (TerL), two proteins often used as markers in phylogenetic analysis. As seen in Figure 3, *Achromobacter* phage AchV4 formed a discrete clade in both resulting trees, indicating that it has no close relatives among sequenced bacterial viruses.

Nevertheless, on both trees, AchV4 clustered with *Pseudomonas syringae* phage Phobos (MN478374) [57], *Pseudomonas* phage PspYZU01 (KY971609), and *Klebsiella* phage YMC16/01/N133_KPN_BP (MF476925), suggesting that these three phages are somewhat related to AchV4. Notably, both BLASTp analysis and the phylogenetic trees obtained indicated that, among sequenced *Achromobacter* viruses, only bacteriophage phiAxp-2 (NC_029106) [10] is distantly related to AchV4.

**Figure 4:**
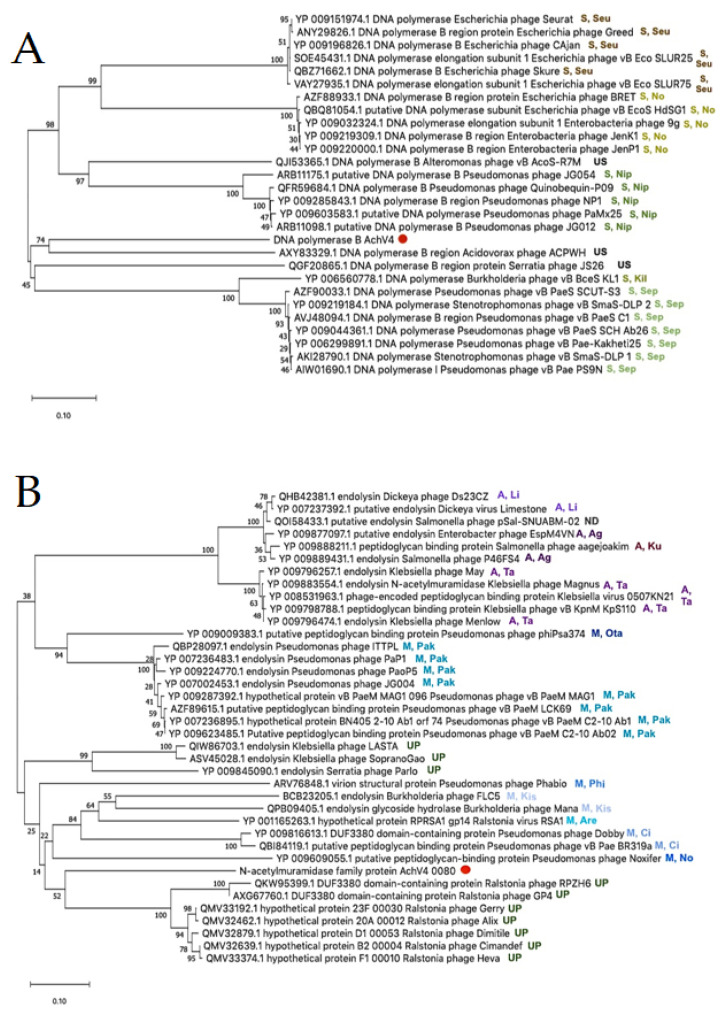
Phylogenetic analysis. Neighbor-joining tree analysis based on the ClustalW alignment of: DNA polymerase (**A**) and phage endolysin (**B**) sequences. The percentage of replicate trees in which the associated taxa clustered together in the bootstrap test (500 replicates) is shown next to the branches. Evolutionary analyses were conducted in MEGA X. US—unclassified siphovirus; UM—unclassified myovirus; UP—unclassified podovirus; S, Seu—*Siphoviridae*, *Seuratvirus*; S, No—*Siphoviridae*, *Nonagvirus*; S, Nip—*Siphoviridae*, *Nipunavirus*; S, Kil—*Siphoviridae*, *Kilunavirus*; S, Sep—*Siphoviridae*, *Septimatrevirus* M, Ota—*Myoviridae*; *Otagovirus*; M, Pak—*Myoviridae*, *Pakpunavirus*; M, Phi—*Myoviridae*, *Phikzvirus*; M, Kis—*Myoviridae*, *Kisquattuordecimvirus*; M, Are—*Myoviridae*, *Aresaunavirus*; M, Ci—*Myoviridae*, *Citexvirus*; M, No—*Myoviridae*, *Noxifervirus*; A, Li—*Ackermannviridae*, *Limestonevirus*; A, Ag—*Ackermannviridae*, *Agtrevirus*; A, Ku—*Ackermannviridae*, *Kuttervirus;* A, Ta—*Ackermannviridae*, *Taipeivirus*; ND—not determined. AchV4 is indicated by a red dot.

To investigate whether DNA metabolism and lysis genes follow the same evolutionary trajectory as the structural module, phylogenetic analysis was also performed on DNA polymerase and endolysin sequences (Figure 4).

From the phylogenetic incongruence observed, it is evident that each AchV4 genome module has its own evolutionary history. The latter result is in keeping with a number of studies suggesting that siphophage genomes are mosaics of genes from various sources, including other phages and their hosts [58,59]. Indeed, as seen in Figure 3 and Figure 4, while the closest relatives of AchV4 TerL, MCP, and DNA polymerase are, in most cases, encoded by siphophages, the closest homologues of the putative AchV4 endolysin are found exclusively in podo- and myoviruses.

**Figure 5:**
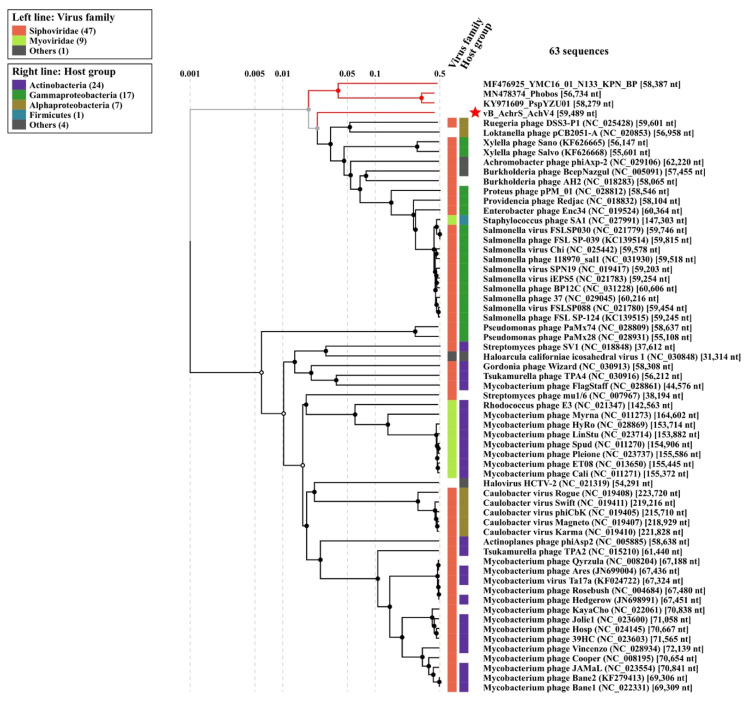
Phylogenetic analysis based on genome-wide sequence similarities computed by tBLASTx. The proteomic tree based on the complete genome sequences of phages with S_G_ values to AchV4 ranging from 0.1581 (Phobos) to 0.05 (Mycobacterium phage JAMaL). The figure was generated by ViPTree.

The remote relationship of AchV4 with bacteriophages Phobos, PspYZU01, YMC16/01/N133_KPN_BP, and phiAxp-2 was also demonstrated by the proteome-based tree generated using ViPTree web-service (Figure 5). Notably, since the Virus-Host database used by the ViPTree does not contain the genome sequences of Phobos, PspYZU01, and YMC16/01/N133_KPN_BP, those were added to the query along with the genome of AchV4.

**Figure 6:**
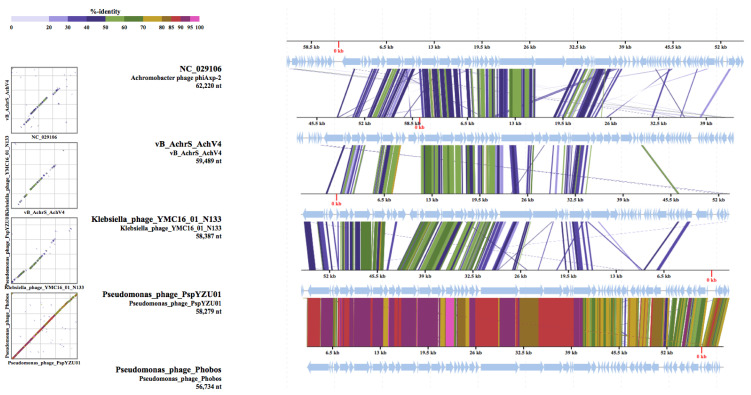
Genome alignment of AchV4, phiAxp-2, Phobos, PspYZU01, and YMC16/01/N133_KPN_BP. All tBLASTx alignments are represented by colored lines between two genomes. Color scale represents the tBLASTx percent identity. The figure was generated by ViPTree.

As seen in Figure 5, AchV4 is indeed distantly related to the four phages listed above; however, the obtained S_G_ scores (Table S5) indicate low protein sequence similarity, which, as seen in Figure 6, is mostly based on structural proteins.

The latter observation was supported by the results of CoreGene 5.0 analysis, which showed that AchV4 shares 26 (32%), 23 (28%), 24 (29%), and 28 (34%) of its proteins with phiAxp-2, Phobos, PspYZU01, and YMC16/01/N133_KPN_BP, respectively (Table S6).

#### Comparison with Sequenced *Achromobacter* Phages

**Figure 7:**
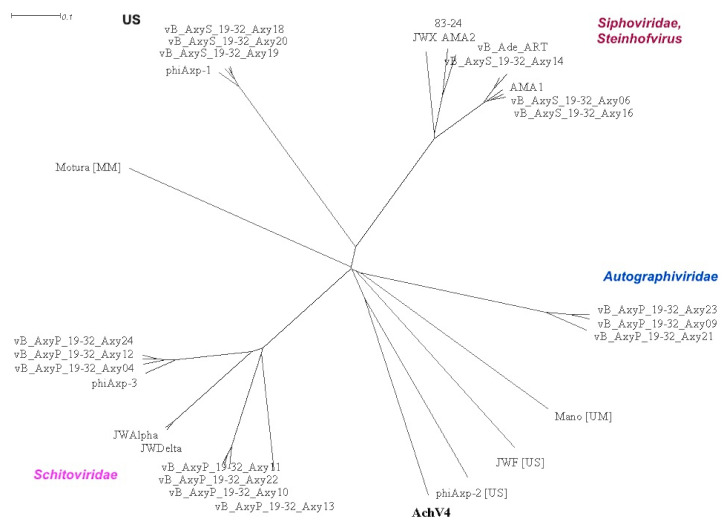
Splitstree representation of the unrooted proteomic tree generated by ViPTree web-service based on the genome-wide similarity relationships between *Achromobacter* bacteriophages. Purple, phages of the family *Schitoviridae*; red, siphoviruses that belong to the genus Steinhofvirus; blue, phages from the family Autographiviridae. UM—unclassified myovirus; MM—family *Myoviridae*, genus Mieseafarmvirus; US—unclassified siphoviruses.

There are currently 29 annotated *Achromobacter* phage genome sequences available in the NCBI database (Table S7). To define the relationship between AchV4 and all other known *Achromobacter* viruses, a proteomic tree based on genome-wide sequence similarities computed by tBLASTx was also generated, and the genome-based distance matrix calculator (available at http://enve-omics.ce.gatech.edu/g-matrix/, accessed on 15 January 2021) was used to estimate all-vs.-all distances between these phages. Based on ANI and AAI values (Figure S4), the genome of AchV4, together with those of phiAxp-2, JWF, Mano, and Motura, is unique among *Achromobacter* viruses. The same conclusion could be drawn from the proteomic tree obtained (Figure 7, Figure S5). As seen in Figure 7, *Achromobacter* viruses represent fourteen distinct lineages that group into four distinct clusters and five singletons: three siphoviruses (AchV4, phiAxp-2, and JWF) and two myoviruses (Mano and Motura).

Of note, a very similar clustering was observed (Figure S6) on the tree obtained using the BLAST-based phylogenetics framework VICTOR [39], which grouped 30 *Achromobacter* phages analyzed into 19 species, 9 genera, and 3 families.

Out of the 30 *Achromobacter*-infecting viruses sequenced to date, 15 belong to the family *Siphoviridae*, AchV4 included. Although all of these viruses have a canonical virion structure common to phages with siphoviral morphologies, comparative genomics analysis indicated that the genome structure of AchV4 significantly differs from that of other *Achromobacter* siphoviruses (Figure S7).

At a glance, the AchV4 virion assembly genes, which comprise the most conserved module in all siphoviruses, are positioned in the same order and orientation as those of phiAxp-2. Upon closer inspection, however, it is evident that the structural module of these two phages differs profoundly over the tail assembly region, and with respect to a number of putative genes of mostly unknown function interspersed among structural genes. Among the many differences observed between the genome of AchV4 and those of other *Achromobacter* siphoviruses, the most notable are: (i) the rearrangements within a structural module, (ii) the location of lysis and DNA metabolism genes, (iii) the number and location of putative genes of unknown function, and (iv) the presence of the lysogeny module. Although two *Achromobacter* siphoviruses, JWX and JWF, have been previously reported to be capable of lysogeny, as seen in Figure S7, only AchV4 codes for identifiable integrase and other lysogeny-related genes.

Taken together, the results of the comparative genomics not only demonstrate the uniqueness of AchV4, whose closest relatives have not yet been identified and characterized, but also highlight the diversity of *Achromobacter* phages.

## 4. Discussion

Achromobacteria are ubiquitous Gram-negative bacilli that belong to the phylum Proteobacteria, class Betaproteobacteria. The organisms have a global distribution, and may be found in water (both fresh and marine), soil, municipal and hospital water supplies as well as human clinical specimens [1,2]. They are important environmental microorganisms capable of degrading a variety of natural, aromatic and xenobiotic compounds [3,4,60,61], and have also been documented as PGPR (plant growth-promoting rhizobacteria), relieving plants from stress conditions by various mechanisms [19,62]. Although *Achromobacter spp*. do not typically cause disease in humans, some members of this genus (e.g., *Achromobacter xylosoxidans* or *Achromobacter denitrificans*) are becoming increasingly associated with lung infections in patients suffering from cystic fibrosis [2,5,18]. As discussed above, within the genus *Achromobacter*, a total of 20 species and 24 genogroups have been recognized thus far; however, little is known about their predators in nature—bacteriophages.

According to the literature, bacteriophages are the most abundant organisms in the biosphere that typically outnumber bacterial populations by at least 10-fold [63,64]. In this context, the fact that 25 out of 30 *Achromobacter* viruses sequenced to date have been isolated on *Achromobacter xylosoxidans* suggests that the phage community has been predominantly focused on clinically relevant hosts, which in turn suggests that *Achromobacter* phage biodiversity is greatly under-sampled.

In this study, we report the isolation and characterization of *Achromobacter spanius-infecting* bacteriophage AchV4. To the best of our knowledge, AchV4 is the first virus reported to be active on bacteria of this particular species. Comparative genome analysis indicated that AchV4 is a unique virus that shows no nucleotide sequence similarity to any of the other bacteriophages sequenced thus far. AchV4 has been predicted to encode 82 proteins, yet no tRNAs. The latter, in combination with a high G+C content (62.8%, similar to that reported for *Achromobacter spanius*), suggests that the phage is well adapted to the translation machinery of the host, and possibly attests their long-term “relationships”. On the other hand, it has been previously suggested that the virulent phages contain more tRNAs and have a higher A+T content than temperate ones [65]. Bacteriophage AchV4 was lytic against *Achromobacter spanius* isolate RL_4 cells under standard laboratory conditions. However, bioinformatics analysis allowed the identification of the lysogeny cassette in the genome of AchV4, suggesting that this phage, under different conditions and/or in the case of a different host, may be capable of lysogeny. Since in the lysogenic state temperate phages share the same mutational biases as the host [65], lysogeny may be the reason for an observed high G+C content in AchV4. Based on homology searches, 27 of AchV4 gene products have no homologues in phages, whereas the majority of those predicted to have homologues in viruses (34 out of 56), are more similar to bacterial rather than phage-encoded proteins. Thus, the genome of AchV4 is a further example of genomic mosaicism, with different genome segments having distinct evolutionary histories. Based on the literature, phages infecting phylogenetically distant hosts share little genetic information and only a few protein-coding genes with amino acid sequence similarity [66,67]. Yet, the data of comparative genome sequence analysis indicated that bacteriophage AchV4 shares more genes with *Klebsiella phage* YMC16/01/N133_KPN_BP (whose host is phylogenetically distant from *Achromobacter* and belongs to Gammaproteobacteria) than it does with any known *Achromobacter* phage or, in fact, with any other phage that infects Betaproteobacteria.

We also extended our analysis to the sequenced complete genomes of *Achromobacter* phages available in GenBank, to obtain a better view of their relationships with AchV4 and to explore *Achromobacter* phage diversity. Our results indicate that AchV4 is distantly related to one out of 30 sequenced *Achromobacter* viruses, *Achromobacter xylosoxidans* bacteriophage phiAxp-2. Although AchV4 and phiAxp-2 share 26 proteins (mostly those involved in virion morphogenesis), both phages are singletons of the family *Siphoviridae*, and their closest relatives are yet to be isolated/sequenced. For such a small group (30 versus thousands of sequenced *Mycobacterium*, *Lactococcus* or Enterobacteria phage genomes), the diversity of *Achromobacter* viruses is remarkable. These 30 phages differ in their virion morphology, genome length, DNA sequence, G+C content, and, as illustrated by the comparison of 15 *Achromobacter* siphophages, both in their genome organization and in gene content. The 30 *Achromobacter* phages considered here represent fourteen distinct lineages that group into four genetically unrelated clusters and five singletons: three siphoviruses (AchV4, phiAxp-2, and JWF) and two myoviruses (Mano and Motura). Within the clusters, phages shared substantial DNA sequence similarity, whereas no similarity was observed between viruses from different clusters. Such great genome sequence diversity is rather unexpected, given that all of these viruses infect *Achromobacter* (moreover, 25 out of 30 infect the same species) and supposedly should be in genetic communication with each other. However, as discussed above, the number of sequenced *Achromobacter* phages is too small and by no means reflects the nature or complexity of the population of viruses that infect this particular host. Hence, more phage genome sequences are needed, from a diverse array of species, to obtain a more complete understanding of *Achromobacter* viruses, especially considering the prevalence and importance of their host, *Achromobacter* spp.

## 5. Conclusions

In this work, we characterized a novel bacteriophage, AchV4, the first virus reported to be active on *Achromobacter spanius*. The data presented here indicate that AchV4 is a singleton siphovirus that belongs to no known genus of tailed phages. Moreover, we show that this particular group of viruses is highly diverse, and emphasize the need for more representative *Achromobacter* phage sequencing data.

## Figures and Tables

**Table 1:** Structural AchV4 proteins identified by MS.

Gene	Putative Function	MW (KDa)	Peptide Count	Sequence Coverage (%)
AchV4_0004	Portal protein	61.537	35	64.69
AchV4_0005	Prohead protease	45.031	23	47.42
AchV4_0006	Head decoration protein	14.105	8	91.79
AchV4_0007	Major capsid protein	38.315	65	93.95
AchV4_0008	Virion structural protein	12.299	24	82.46
AchV4_0009	Head closure protein	14.922	12	81.06
AchV4_0010	Neck protein	22.423	17	74.63
AchV4_0012	Major tail protein	28.341	31	75.77
AchV4_0015	Tape measure protein	167.818	232	83.54
AchV4_0016	Virion structural protein	36.358	12	61.79
AchV4_0017	Virion structural protein	33.351	20	61.86
AchV4_0019	Virion structural protein	57.374	24	50.58
AchV4_0020	Virion structural protein	35.315	27	61.76
AchV4_0021	Virion structural protein	6.989	2	53.13
AchV4_0023	Virion structural protein	97.678	53	69.37
AchV4_0024	Putative tail fiber protein	26.079	13	90.83
AchV4_0025	Virion structural protein	35.935	22	75.08
AchV4_0026	Virion structural protein	37.575	14	35.51

Genes with no amino acid sequence similarity to known viral structural proteins are shaded in grey.

## Data Availability

The complete genome sequence of *Achromobacter* phage ArV1 is available at the NCBI, accession number MW269554. The raw reads may be found at the NCBI Sequence Read Archive (SRA), accession number SRX8646550.

## Supplementary Materials

The following are available online at https://www.mdpi.com/1999-4915/13/3/374/s1. Table S1: bacterial strains used in this study. Figure S1: plaques formed by AchV4 on a lawn of *Achromobacter spanius* RL_4. Figure S2: growth parameters of AchV4. Figure S3: effect of proteinase K and periodate treatment on the adsorption of AchV4. Table S2: protein-coding AchV4 genes. Table S3: list of regulatory sequences predicted by PhagePromoter. Table S4: Rho-independent terminators predicted by ARNold. Table S5: phage genomes used for the construction of proteome-based tree. Table S6: proteins shared between AchV4 and related phages as identified by CoreGenes 5.0. Table S7: *Achromobacter* phages with completely sequenced genomes. Figure S4: the average nucleotide and amino acid identities between *Achromobacter* phage genomes. Figure S5: proteomic tree generated based on the genome-wide similarity relationships between 30 *Achromobacter* bacteriophages and related viruses from the Virus-Host database. Figure S6: Splitstree representation of the unrooted phylogenomic GBDP tree. Figure S7. Schematic representation of the genome organization of *Achromobacter* siphoviruses.
