# Supplementary material for: Analysis of a Novel Bacteriophage vB_AchrS_AchV4 Highlights the Diversity of Achromobacter Viruses
Source: Viruses. 2021 Feb 27;13(3):374. doi: 10.3390/v13030374 (PMC7996906; doi:10.3390/v13030374)
Supplement: Supplementary file 1 [file viruses-13-00374-s001.pdf]

Supplementary Material for:
Viruses

Analysis of a Novel Bacteriophage vB_AchrS_AchV4 Highlights the Diversity of *Achromobacter* Viruses

Laura Kaliniene[1*], Algirdas Noreika[1], Algirdas Kaupinis[2], Mindaugas Valius[2], Edvinas Jurgelaitis[1], Justas Lazutka[3], Rita Meškienė[1], Rolandas Meškys[1]

1 Department of Molecular Microbiology and Biotechnology, Institute of Biochemistry, Life Sciences Center , Vilnius University, Saulėtekio av. 7, LT- 10257 Vilnius, Lithuania; laura.kaliniene@bchi.vu.lt (L.K.); algirdas.noreika@gmc.vu.lt (A.N.); edvinas.jurgelaitis@chgf.stud.vu.lt (E.J.); rita.meskiene@bchi.vu.lt (Ri.M.); rolandas.meskys@bchi.vu.lt (R.M.)

2 Proteomics Centre, Institute of Biochemistry, Life Sciences Centre, Vilnius University, Saulėtekio av. 7, LT- 10257 Vilnius, Lithuania; algirdas.kaupinis@gf.vu.lt (A.K.); mindaugas.valius@bchi.vu.lt (M.V.)

3 Department of Eukaryote Gene Engineering, Institute of Biotechnology, Life Sciences Center, Vilnius University, Saulėtekio av. 7, LT- 10257 Vilnius, Lithuania; justas.lazutka@bti.vu.lt (J.L)

***Correspondence: laura.kaliniene@bchi.vu.lt; Tel.: +370-5-2234384**

Table S1. Bacterial strains used in this study.

Strain	Relevant characteristics	Source or reference (16S rRNA accession number)
***Achromobacter spanius* RL_4**	Environmental isolate	Laboratory collection, (MT670403)
Achromobacter sp. RL_8	Environmental isolate	Laboratory collection, (MT671117)
Achromobacter piechaudii/marplatensis	Environmental isolate	Prof. E. Suziedeliene [22]
Caballeronia glathei, DSM 50014 (Zolg and Ottow, 1975) Dobritsa and Samadpour, 2016	Type strain	DSMZ
Burkholderia sp. MAK1	Environmental isolate	Laboratory collection, (KU195413) [23]
Alcaligenes eutrophus AE	Environmental isolate	Laboratory collection
Duganella zoogloeoides KR2-3	Environmental isolate	Laboratory collection, (MN752691)
Citrobacter freundii		Prof. E. Suziedeliene
Enterobacter cloacae		Prof. E. Suziedeliene
Erwinia carotovora 8982		Prof. E. Suziedeliene
Escherichia coli B^E	sup^0	Dr. L. W. Black
Escherichia coli BL21 (DE3)	F^- dcm ompT hsdS(rB^-mB^-) gal λ(DE3)	Avidis
Escherichia coli DH5α	F^- endA1 glnV44 thi-1 recA1 relA1 gyrA96 deoR nupG Φ80dlacZΔM15 Δ(lacZYA-argF)U169, hsdR17(r_K^- m_K^+), λ–	Pharmacia
Escherichia coli DH10β	F^- endA1 recA1 galE15 galK16 nupG rpsL ΔlacX74 Φ80lacZΔM15 araD139 Δ(ara,leu)7697 mcrA Δ(mrr-hsdRMS-mcrBC) $λ^-$	Invitrogen
Klebsiella pneumoniae 279		Prof. E. Suziedeliene
Pseudomonas aeruginosa PAO1		Prof. E. Suziedeliene
Salmonella enterica ser. Typhimurium 292		Prof. E. Suziedeliene

- AchV4-sensitive strain is in bold.

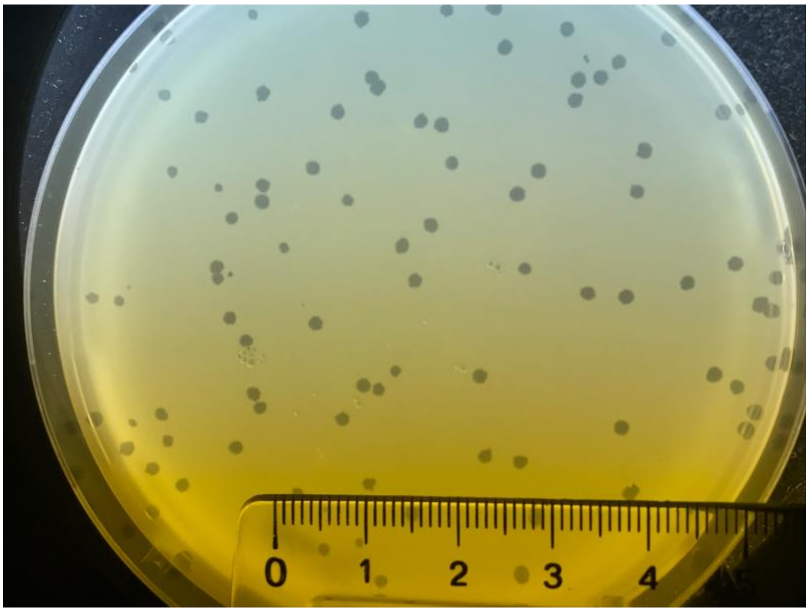

Figure S1. Plaques formed by AchV4 on a lawn of *Achromobacter spanius* RL_4. The ruler is in cm.

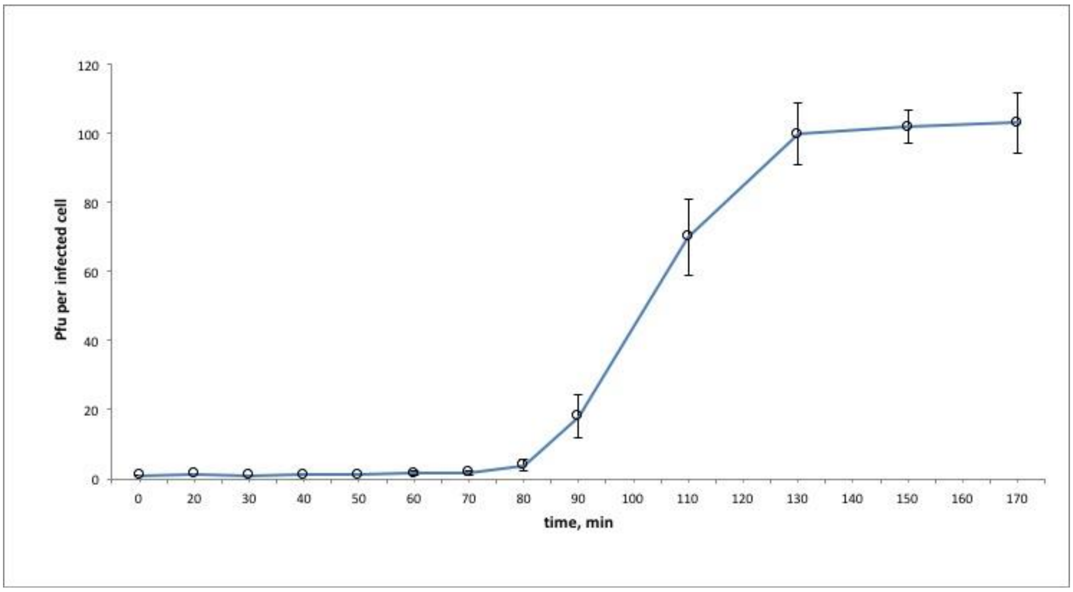

Figure S2. Growth parameters of AchV4. The latent period of AchV4 is ~80 min, and the burst size of one lytic cycle is 100 pfu per infected cell. Three biological replicates performed. The figure was generated using Microsoft Excel.

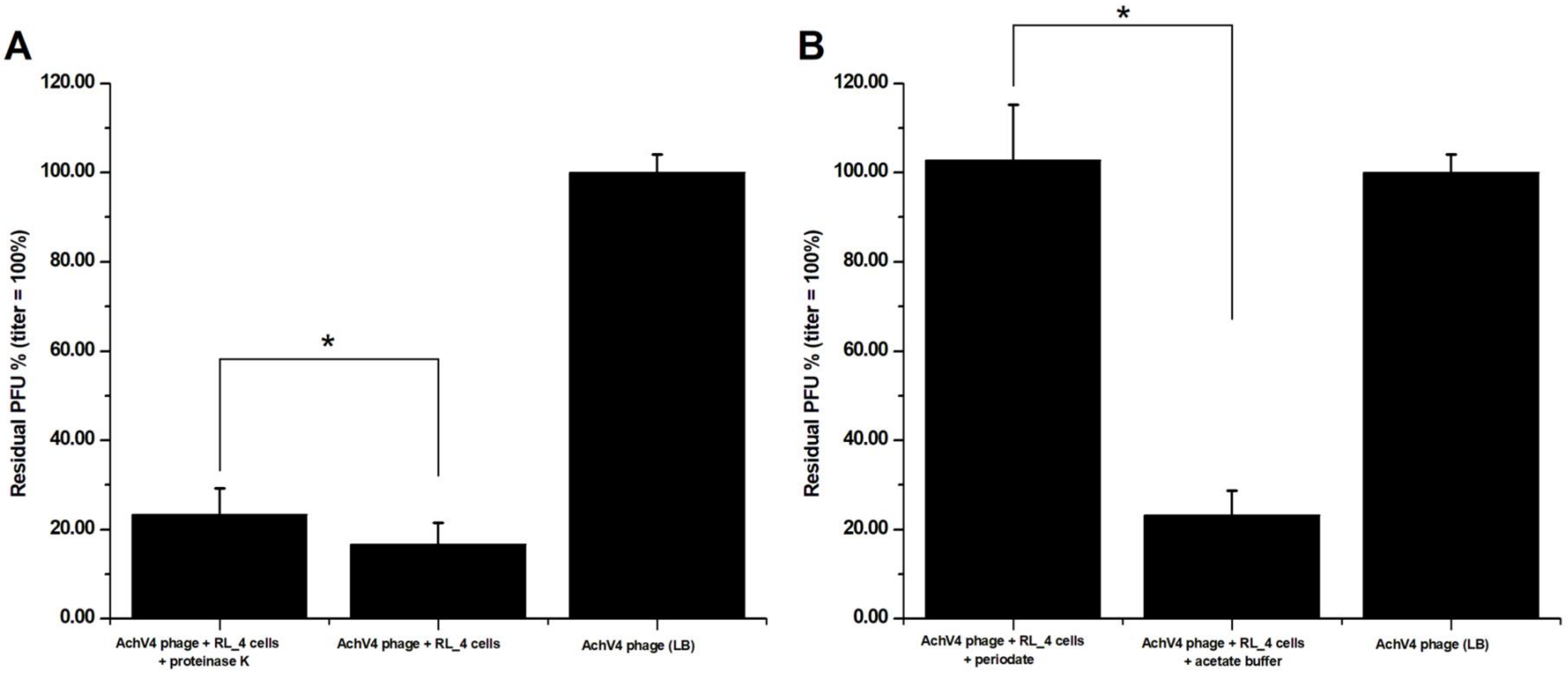

Figure S3. Effect of proteinase K and periodate treatment on the adsorption of AchV4. (A) Effect of proteinase K treatment on the adsorption of AchV4 to RL_4 cells. (B) Effect of periodate treatment on the adsorption of AchV4 to RL_4 cells. The control (AchV4 phage (LB), AchV4 phage + RL_4 cells, and AchV4 phage + RL_4 cells + acetate buffer), and treatment (AchV4 phage + RL_4 cells + proteinase K, for proteinase K treatment and AchV4 phage + RL_4 cells + periodate, for periodate treatment) groups were tested for adsorptions as indicated on the x axes. Error bars denote statistical variations. Significance was determined by one Student t test for comparison between the treated group and the WT group. *, $P < 0.05$. The figure was generated using Origin v6.0.

Table S2. Protein-coding AchV4 genes.

Locus_tag	Length aa	Product (conserved domain/protein family, accession Nr.)	Top BLASTp match<br>E value<br>identity, %<br>coverage, %	Reference phage protein<br>identity, %<br>coverage, %
AchV4_0001	241	Terminase small subunit<br><br>(DUF1441, PF07278)	Terminase small subunit [Bacteriophage Phobos] 2.40E-56<br>45.8<br>93.3	[illegible]
AchV4_0002	694	Terminase large subunit<br><br>(Terminase_GpA, PF05876)	Phage terminase large subunit family protein [Bradyrhizobium sp. WSM3983]<br>0<br>61.5<br>99.3	Terminase large subunit [Bacteriophage Phobos]<br>0<br>60.4<br>96.4
AchV4_0003	69	Head-to-tail joining protein<br><br>(gpW, PF02831)	Hypothetical protein [unclassified Labrenzia]<br><br>6.10e-19<br>54.1<br>94.87	Head-tail joining protein [Synechococcus virus S-ESS1]<br>8.60e-17<br>60.9<br>80.8
AchV4_0004	556	Portal protein<br><br>(Phage_portal_2; PF05136)	Phage portal protein [Bradyrhizobium sp. WSM3983]<br>0<br>61.4<br>97.8	Portal protein [Ruegeria phage DSS3-P1]<br>0<br>56.5<br>98.2
AchV4_0005	427	Prohead protease<br><br>(Peptidase_S49, PF01343)	S49 family peptidase [Sinorhizobium meliloti]<br><br>8.65e-139<br>54.3<br>95.5	Prohead protein [Bacteriophage Phobos]<br>3.82e-116<br>43.3<br>99.1
AchV4_0006	135	Head decoration protein<br><br>(Head_decoration_D, IPR004195)	Head decoration protein [Gemmobacter caeni]<br><br>9.90E-10<br>47.0<br>61.2	Putative head decorative protein [Rhizobium phage vB_RleM_PPF1]<br><br>1.26e-07<br>37.6<br>91.0

AchV4_0007	348	Major capsid protein<br><br>(Phage_cap_E, cl20258)	Minor capsid protein E [Pseudomonas phage PspYZU01]<br>2.55e-139<br>57.1<br>99.7	[illegible]
AchV4_0008	115	Virion structural protein	-----	-----
AchV4_0009	133	Head closure protein	Hypothetical protein ADP64_000020 [Achromobacter phage phiAxp-2]<br>2.90e-29<br>47.5<br>97.7	-----
AchV4_0010	206	Neck protein	Hypothetical protein AH2_00059 [Burkholderia phage vB_BceS_AH2]<br>4.47e-83<br>60.7<br>98.1	Neck protein [Providencia phage vB_PreS-PatoteraRojo]<br>7e-40<br>44.9<br>88.0
AchV4_0011	164	Minor tail protein	Hypothetical protein [Mesorhizobium sediminum]<br><br>7.60e-48<br>51.5<br>100	Minor tail protein [Klebsiella phage YMC16/01/N133_KPN_BP]<br>7.98e-42<br>46.4<br>99.4
AchV4_0012	261	Major tail protein	Hypothetical protein AH2_00057 [Burkholderia phage vB_BceS_AH2]<br>9.19e-106<br>61.2<br>95.4	Major tail protein [Klebsiella phage YMC16/01/N133_KPN_BP]<br>1.26e-85<br>51<br>98.1
AchV4_0013	144	Tail assembly protein	Hypothetical protein [Mesorhizobium sediminum]<br><br>3.26e-20<br>37.6<br>87.4	Tail assembly chaperone [Pectobacterium phage MA11]<br>7.52e-11<br>33.6<br>97.9
AchV4_0014	71	Tail protein	Tail protein [Burkholderia phage vB_BceS_AH2]<br>5.69e-19<br>60.6<br>94.3	[illegible]
AchV4_0015	1587	Tape measure protein	Tape measure protein [Rhizobium sp. RU20A]	Tail length tape-measure protein [Bacteriophage Phobos]

		(Caudovirus_tape_meas_ N, IPR013491)	0<br>33.0<br>99.9	8.82e-107<br>31.3<br>63.4
AchV4_0016	336	Virion structural protein	Hypothetical protein DI533_20285 [Rhodobacter sphaeroides]<br>5.00e-78<br>42.8<br>99.7	Virion structural protein [Pseudomonas phage Fc22]<br>7.65e-42<br>32.0<br>96.6
AchV4_0017	313	Virion structural protein	Hypothetical protein E6R03_08550 [Hyphomicrobiaceae bacterium]<br>2.27e-37<br>35.4<br>86.9	Virion structural protein [Pseudomonas phage Fc22]<br>5.42e-05<br>24.0<br>67.3
AchV4_0018	73	Hypothetical protein	-----	-----
AchV4_0019	517	Virion structural protein	Hypothetical protein DI533_20275 [Rhodobacter sphaeroides]<br>4.36e-123<br>40.7<br>97.9	Capsid and scaffold protein [Bacteriophage Phobos]<br>2.33e-52<br>31.0<br>76.2
AchV4_0020	284	Virion structural protein<br><br>(Phage_BR0599, cl10710)	Phage BR0599 family protein [Sinorhizobium fredii]<br><br>9.81e-76<br>44.1<br>100	Putative structural protein [Pseudomonas phage PAE1]<br>6.22e-40<br>35.0<br>97.5
AchV4_0021	65	Virion structural protein	Hypothetical protein [Sphingobium barthaii]<br><br>1.34e-24<br>68.9<br>95.3	Putative virion structural protein [Stenotrophomonas phage DLP4]<br>4.69e-14<br>47.6<br>98.4
AchV4_0022	77	Putative tail assembly protein	Hypothetical protein [Halomonas halophila]<br><br>1.39e-11<br>50.9<br>69.7	Tail assembly protein [Bordetella phage FP1]<br>9.97e-11<br>44.6<br>73.7
AchV4_0023	889	Virion structural protein<br><br>(Phage-tail_3, PF13550)	Hypothetical protein DI533_20255 [Rhodobacter sphaeroides]<br>5.79e-168	Putative tail protein [Ruegeria phage DSS3-P1]<br><br>2.05e-101<br>36.3

			39.9<br>79.5	65.8.3
AchV4_0024	241	Putative tail fiber protein<br><br>(DUF2793, cl12557; Fibritin, SSF58046)	DUF2793 domain-containing protein [Rhizobium leguminosarum]<br>2.16e-15<br>29.1<br>92.9	Putative tail fiber protein [Mesorhizobium phage Cp1R7A-A1]<br>2.27e-10<br>41.3<br>42.5
AchV4_0025	334	Virion structural protein	Hypothetical protein [Burkholderia ubonensis]<br><br>1.52e-65<br>39.0<br>100	Hypothetical protein [Salmonella phage SAP012]<br>3.92e-62<br>37.8<br>99.4
AchV4_0026	353	Virion structural protein	Hypothetical protein Lu11_0243 [Pseudomonas phage Lu11]<br>4.91e-27<br>33.9<br>61.7	----
AchV4_0027	378	Hypothetical protein	-----	-----
AchV4_0028	579	Hypothetical protein	Hypothetical protein pEpSNUABM08_36 [Erwinia phage pEp_SNUABM_08]<br>6.34e-08<br>23.6<br>70.6	[illegible]
AchV4_0029	134	Putative holin<br><br>(Phage_holin_3_1, PF05106)	Phage holin, lambda family [Plesiomonas shigelloides]<br>1.91e-21<br>39.6<br>83.5	Putative holin [Aeromonas virus phiO18P]<br>4.61e-15<br>42.2<br>81.96
AchV4_0030	80	Hypothetical protein	Hypothetical protein [Oxalobacteraceae bacterium]<br>3.22e-10<br>44.1<br>86.1	Hypothetical protein [Pseudomonas phage Persinger]<br>3.85e-09<br>38.2<br>86.1
AchV4_0031	204	Hypothetical protein<br><br>(DUF3310, PF11753)	Hypothetical protein KPNN133_054 [Klebsiella phage YMC16/01/N133_KPN_BP]<br>1.30e-20<br>42.9<br>49.8	[illegible]
AchV4_0032	72	Hypothetical protein	-----	-----

AchV4_0033	71	Hypothetical protein<br><br>(WH-like_DNA-bd_sf, IPR036388)	helix-turn-helix domain-containing protein [Achromobacter phage Mano]<br>6.35e-05<br>48.0<br>71.4	[illegible]
AchV4_0034	75	Hypothetical protein	-----	-----
AchV4_0035	77	Hypothetical protein	-----	-----
AchV4_0036	309	Recombination-associated protein RdgC<br><br>(RdgC, IPR007476)	Recombination-associated protein RdgC [Achromobacter denitrificans]<br>2.68e-143<br>65.5<br>98.7	DNA recombination-dependent growth factor C [Acidovorax phage ACP17]<br>8.07e-55<br>34.1<br>97.7
AchV4_0037	109	Hypothetical protein	-----	-----
AchV4_0038	238	dUTPase<br><br>(NTP-PPase_dUTPase, cl16941)	Hypothetical protein [Burkholderia gladioli]<br>6.29e-33<br>41.9<br>90.4	Hypothetical protein [Bacteriophage sp.]<br>1.53e-19<br>29.7<br>90.3
AchV4_0039	219	Hypothetical protein<br><br>(NT_sf, IPR043519)	Hypothetical protein [Burkholderia gladioli]<br><br>6.29e-33<br>41.9<br>90.4	--------
AchV4_0040	67	Hypothetical protein	-----	-----
AchV4_0041	86	Hypothetical protein	Hypothetical protein C0J09_13980 [Bordetella avium]<br>4.70e-26<br>54.9<br>96.5	-----
AchV4_0042	86	Hypothetical protein	Hypothetical protein [Bordetella phage FP1]<br>9.21e-06<br>61.5<br>60.3	[illegible]
AchV4_0043	125	Hypothetical protein	Hypothetical protein [Achromobacter sp. DH1f]<br>9.69e-44<br>75.3<br>71.8	-----
AchV4_0044	154	Hypothetical protein	Hypothetical protein [Achromobacter deleyi]	-----

			4.79e-15<br>72.0<br>32.5	
AchV4_0045	113	Hypothetical protein	Hypothetical protein [Burkholderia gladioli]<br>1.42e-08<br>48.6<br>62.1	-----
AchV4_0046	124	Hypothetical protein	-----	-----
AchV4_0047	216	Hypothetical protein	-----	-----
AchV4_0048	106	Hypothetical protein	Hypothetical protein ADP64_000071 [Achromobacter phage phiAxp-2]<br>7.10e-06<br>38.2<br>84.8	[illegible]
AchV4_0049	103	Hypothetical protein	Hypothetical protein [Dechloromonas sp. H13]<br>3.39e-18<br>43.0<br>98.0	hypothetical protein [Pseudomonas phage PMBT3]<br>6.45e-13<br>38.5<br>98.1
AchV4_0050	60	Hypothetical protein	-----	-----
AchV4_0051	150	Hypothetical protein	Hypothetical protein [Alcaligenes faecalis]<br>9.69e-19<br>41.1<br>75.2	-----
AchV4_0052	76	Hypothetical protein	Hypothetical protein EOS77_02330 [Mesorhizobium sp.]<br>3.90e-11<br>47.9<br>93.3	Hypothetical protein CcrSC_gp029 [Caulobacter phage CcrSC]<br>8.52e-08<br>52.0<br>66.7
AchV4_0053	242	Hypothetical protein	hypothetical protein [Burkholderia pseudomallei]<br>1.67e-49<br>47.4<br>69.7	hypothetical protein UFOVP400_38 [uncultured Caudovirales phage]<br>5.60e-35<br>43.8<br>68.1
AchV4_0054	161	Hypothetical protein	Hypothetical protein [Ralstonia solanacearum]<br>6.93e-48<br>50.6	hypothetical protein LAV_00211 [Sphingobium phage Lacusarx]<br>1.50e-37<br>51.7<br>64.3

			86.0	
AchV4_0055	87	Hypothetical protein	-----	-----
AchV4_0056<br>(**)	232	Hypothetical protein	Hypothetical protein ADP64_000072 [Achromobacter phage phiAxp-2]<br>1.56e-15<br>43.3<br>41.4	
AchV4_0057	135	Hypothetical protein	-----	-----
AchV4_0058<br>(**)	104	Hypothetical protein	Hypothetical protein RG2014_066 [Delftia phage RG-2014]<br>5.77e-23<br>46.5<br>98.1	
AchV4_0059	84	Hypothetical protein	-----	-----
AchV4_0060	125	Hypothetical protein	Hypothetical protein [Pseudomonas phage Lana]<br>7.86e-07<br>62.2<br>29.8	
AchV4_0061	105	Hypothetical protein	Hypothetical protein [Diaphorobacter sp. NR2-3-3-1]<br>1.24e-06<br>61.9<br>40.0	-----
AchV4_0062	96	Hypothetical protein	-----	-----
AchV4_0063	63	Hypothetical protein	-----	-----
AchV4_0064	114	Hypothetical protein<br>(DUF982, IPR010385)	DUF982 domain-containing protein [Phyllobacterium sp. CL33Tsu]<br>1.48e-42<br>67.6<br>93.8	-----
AchV4_0065	64	Hypothetical protein	Hypothetical protein FD152_392 [Xanthobacteraceae bacterium]<br>2.22e-11<br>49.2<br>100	Hypothetical protein POI1126_07 [Ochrobactrum phage POI1126]<br>3.44e-07<br>43.4<br>82.5
AchV4_0066	76	Hypothetical protein	-----	-----
AchV4_0067	88	Hypothetical protein	-----	-----
AchV4_0068	119	Hypothetical protein	Hypothetical protein [Bacteriophage Phobos]<br>1.52e-06<br>29.9	

			98.3	
AchV4_0069	64	Hypothetical protein	Hypothetical protein B0184_08395 [Haemophilus paraphrohaemolyticus]<br><br>5.79e-04<br>38.0<br>81.4	Hypothetical protein KPNN133_066 [Klebsiella phage YMC16/01/N133_KPN_BP]<br>9.21e-04<br>34.5<br>62.8
AchV4_0070	882	DNA primase/polymerase<br><br>(DNA_primase/pol_bifunc_N, IPR015330; PriCT_2, IPR014819; Phage/plasmid_primase_P4_C, PF08706; P-loop_NTPase, SSF52540)	Hypothetical protein E6R03_05715 [Hyphomicrobiaceae bacterium]<br>1.40e-110<br>37.8<br>65.0	Putative DNA primase [Prokaryotic dsDNA virus sp.]<br><br>6.33e-80<br>27.8<br>93.9
AchV4_0071	90	Transcriptional regulator<br><br>(YdaS_antitoxin, IPR031856; HTH_XRE Superfamily, cl22854)	Hypothetical protein KPNN133_069 [Klebsiella phage YMC16/01/N133_KPN_BP]<br>1.96e-15<br>44.0<br>84.3	□
AchV4_0072	207	Hypothetical protein	-----	-----
AchV4_0073	388	Hypothetical protein<br><br>(DUF2800, IPR021229)	Hypothetical protein PspYZU01_57 [Pseudomonas phage PspYZU01]<br>4.31e-85<br>40.9<br>91.4	□
AchV4_0074	201	Putative DNA replication protein<br><br>(NA-bd_OB-fold, IPR012340; DUF2815, pfam10991)	hypothetical protein [Bacteriophage Phobos]<br>2.76e-49<br>50.3<br>84.5	□
AchV4_0075	654	DNA polymerase B<br><br>(DNA/RNA_pol_sf, SSF56672; DNA_pol_palm_dom_sf, IPR023211)	Hypothetical protein [Pseudomonas aeruginosa]<br>0<br>50.9<br>96.8	DNA polymerase B region [Acidovorax phage ACPWH]<br>0<br>47.3<br>97.9
AchV4_0076	88	Hypothetical protein	Hypothetical protein DI537_20480	Hypothetical protein LOKG_00017 [Loktanella phage pCB2051-A]

		(VRR_NUC, IPR014883;<br>Restrct_endonuc-II-like, IPR011335)	[Pseudomonas stutzeri]<br><br>2.97e-24<br>56.0<br>95.4	1.06e-23<br>53.4<br>100
AchV4_0077	516	DNA helicase<br><br>(SNF2_N, PF00176;<br>Helicase_ATP-bd, IPR014001)	DEAD/DEAH box helicase [unclassified Labrenzia]<br><br>1.44e-160<br>50.0<br>99.0	Hypothetical protein LOKG_00018 [Loktanella phage pCB2051-A]<br>1.89e-141<br>46.0<br>94.8
AchV4_0078	222	Putative helix-turn-helix transcriptional regulator<br><br>(HTH_XRE, cl22854)	Putative HTH-domain containing protein [Klebsiella phage YMC16/01/N133_KPN_BP]<br>2.43e-96<br>57.9<br>95.7	
AchV4_0079	78	Putative excisionase	Putative excisionase [Klebsiella phage YMC16/01/N133_KPN_BP]<br>3.55e-30<br>76.2<br>81.8	
AchV4_0080	194	N-acetylmuramidase family protein<br><br>(Muramidase, IPR024408)	Hypothetical protein B2_00004 [Ralstonia phage Cimandef]<br>1.45e-71<br>58.9<br>99.5	Endolysin [Klebsiella phage LASTA]<br>1.06e-61<br>50.0<br>98.7
AchV4_0081	405	Arm DNA-binding domain-containing integrase<br><br>(Arm-DNA-bind_3, pfam13356;<br>INT_P4_C, cd00801;)	Integrase arm-type DNA-binding domain-containing protein [Azorhizobium sp. AG788]<br>3.99e-180<br>62.9<br>98.8	Integrase [Pseudomonas phage phiAH14b]<br><br>5e-107<br>43.3<br>99.0
AchV4_0082	67	AlpA family transcriptional regulator<br><br>(Phage_AlpA, PF05930)	AlpA family transcriptional regulator [Thalassobius autumnalis]<br>7.53e-15<br>52.5<br>92.4	transcriptional regulator [Enterobacteria phage P4]<br>2.72e-11<br>42.9<br>95.5

- Predicted AchV4 proteins that have no homologues in phages are shaded in grey.

Table S3. List of regulatory sequences predicted by PhagePromoter

	Strand	Positions	Promoter Sequence	Type	Scores
1	-	(1080..1100)	CCAGGGGGCCGATCTCCTTCG	phage	0.542
2	+	(5473..5493)	CCGGGACACCGAAGCGCTGAC	phage	0.646
3	-	(7405..7425)	CAGCGGGGCCGACTGGCTCAT	phage	0.721
4	-	(9455..9483)	TTGCCATGGTCTACCTCTTGGTTGATAAT	host	0.875
5	-	(15134..15160)	CGCCCAGGGCGTCCTTCCACGACATCG	phage	0.991
6	+	(15540..15572)	CGGCCAGTCTGGCGCAACCCTGAATGCTAAAAT	host	0.935
7	+	(15543..15563)	CCAGTCTGGCGCAACCCTGAA	phage	0.733
8	-	(26105..26127)	TAATTCCTCACACTACCAGGGTT	phage	0.886
9	+	(28323..28349)	CGCCCGCCAACAACTTCGGCCCGAACT	phage	0.994
10	-	(29457..29477)	CCTGGTCGCCGCCAGTCTTCT	phage	0.921
11	-	(32215..32246)	AGCGCCGCCCAGCTCATCGGGCGCGACCTGAA	phage	0.901
12	-	(33271..33293)	TGAATAAAGTCACTATCGAGCAG	phage	0.898
13	-	(33319..33341)	TAATTCGTCTCACGATAAACAAC	phage	0.899
14	+	(33554..33580)	CGTCCTGGCCGTCCTCCGGCGTGAAGC	phage	0.509
15	-	(33817..33837)	GCGGTTCGCCGACTGCCACCT	phage	0.706
16	-	(35170..35199)	CTGAAGGCGCGGCGGAATTGATCGAATACT	host	0.502
17	+	(35575..35601)	TTGACACGGGGCCGGTGTTGTTCTGGT	host	0.609
18	-	(36929..36958)	TTGAGTTTCAACCAAAAGGTGACTTATACT	host	0.904
19	-	(39599..39630)	CGGACCCGGACGAAGTAAAGCGGAATTATTCT	host	0.509
20	+	(41357..41383)	CGGCCATCGTTCACGCGGGCCGTGAAT	phage	0.950
21	-	(43118..43144)	CGGGCACGCCCGCCGCCGTGGTCATTC	phage	0.627
22	-	(43560..43586)	CAACAAGGGGCTACGCCCGCCCTCATC	phage	0.982
23	-	(44634..44654)	CCGGGTTGGCGTCTCCATGAA	phage	0.999
24	+	(46925..46951)	AGGCCACGGTCGACTTCGGCCTCAACC	phage	0.856
25	+	(49184..49204)	ACAGCTTGACGACTTCCTGCG	phage	0.993
26	-	(50693..50713)	GCGGGTTGCGCACCGCCTCCA	phage	0.837
27	+	(51655..51681)	CGGACACGCCCCACGGCAAGGCCAACC	phage	0.740
28	-	(52460..52480)	TCCAGTCGCTGACTTCCTTGA	phage	0.729
29	-	(53435..53455)	CGGGGATGCCGTCTTCCTCCA	phage	0.970
30	+	(54742..54768)	CGACCCTCGCGTTCGCGCCGCCTATAA	phage	0.997

Table S4. Rho-independent terminators predicted by ARNold

5' end position	Program that produced prediction	Strand	Sequence	Free energy of stem-loop region (kcal/mol)
1379	Rnamotif	+	CCGCACGAGGCGCCGCCCACCCGCGGCaTTTTGGCGCTGT	-9.70
5608	Rnamotif	–	GCTTTATCCAGGTCTGCCTGCGCGAAGGCGGGCTTTTGGGTGGGG	-13.40
7513	Rnamotif	+	TCGAACTCGACGCCCCGGTGGGGGCaTTTTTCCAAAGG	-11.60
24084	Rnamotif	–	GGCAGTGGCAAGCGTGGTCGACGTGGCGGCCGCGCcgTTGATGTTCGCG	-14.00
25694	Rnamotif	–	CGTCAGCCTGCGGTTGGCGAGTCACGACCTTTACCTTACCG	-6.60
27115	Rnamotif	–	ATCCCCGTGGGGGTGGTGGTCACACCCGTCACCGTCgTTTTATTGAAGG	-6.00
33295	Both	+	GTCCTTCCTTTTGCGCCGTAGCGCGTTGTTTATCGTG	-6.10
37758	Both	–	CAAATTCAAAGCCCCGCCCCGTGCGGGGTTTCTTTTTGCC	-11.90
37759	Rnamotif	+	GCAAAAAGAAACCCCGCACGGGGCGGGGcTTTGAATTTGGC	-12.50
38883	Rnamotif	+	CTTCGCGCAGCGTCGCGACCGTGAACGCGGCaTGTTTTTTACTG	-9.30
39337	Rnamotif	+	CGGCAAGGCGGGCGTGTTCGGCGGCGCGCTTTTCCATGTGC	-8.20
41859	Rnamotif	–	CCGAATTCCCGCCCGGCCGCGTGCCGGGTTTTTCATGCCC	-11.90
42565	Rnamotif	+	TCAACCTCCAGGCGCGCAAGCGCTTTGCTTTCGGC	-6.90
44951	Rnamotif	–	CCTGACCCCAGCCCCGCCGAGTGCGGGGTTTCTTTTTGCG	-11.90
46372	Both	–	TCCCTAGTTCAGCCCGGCCAGTCCGGGCacTTTTTTGTCCCA	-12.00
52132	Rnamotif	+	ATTGCGTTTCCGGGGACTGCCGAAGGGTGGTCCCCTTTTTCCTTGTG	-12.30
52166	Rnamotif	+	CTTTTTCCTTGTGGGGGCTGTGTTCCCCCGTTTCTCTTCCCG	-9.20
55039	Rnamotif	–	GCCGGGCGGGATCGGCTTGCCGTCCGGGCCGAgcTTTTTGCTGGGT	-10.80
55847	Rnamotif	+	TTCAACAAACGGTTGGATCGGTCAGGCCAGCTTTTTTCAGTAA	-4.30
58915	Rnamotif	–	GTGCCCCGCAGGCCGTGAACGGTGGCGCGGCTTTTGTAGCCCA	-9.80

Table S5. Phage genomes used for the construction of proteome-based tree.

ID	length	name	group	host_group	score	S_G	% mean idt.	% len
**MW269554**	59489	**vB_AchrS_AchV4**	dsDNA	Betaproteobacteria	41339	1	100	99.9
MN478374	56734	P. syringae phage Phobos	dsDNA	Gammaproteobacteria	6305	0.1581	48.7	31
NC_029106	62220	Achromobacter phage phiAxp-2	dsDNA	Betaproteobacteria	6281	0.1519	45.3	35.1
MF476925	58387	Klebsiella phage YMC16/01/N133_KPN_BP	dsDNA	Gammaproteobacteria	6099	0.1517	49.1	29.6
KY971609	58279	Pseudomonas phage PspYZU01	dsDNA	Gammaproteobacteria	5997.5	0.1481	49.5	28.7
NC_018283	58065	Burkholderia phage AH2	dsDNA	Betaproteobacteria	5700.5	0.1379	45.7	30.8
NC_025428	59601	Ruegeria phage DSS3-P1	dsDNA	Alphaproteobacteria	5573	0.1348	46.8	28.9
NC_020853	56958	Loktanella phage pCB2051-A	dsDNA	Alphaproteobacteria	5393	0.1305	47.2	27.4
KF626665	56147	Xylella phage Sano	dsDNA	Gammaproteobacteria	4266.5	0.1032	44.6	23.6
NC_019408	223720	Caulobacter virus Rogue	dsDNA	Alphaproteobacteria	4079.5	0.0987	36.8	22.6
KF626668	55601	Xylella phage Salvo	dsDNA	Gammaproteobacteria	3954	0.0956	43.7	22.7
NC_021783	59254	Salmonella virus iEPS5	dsDNA	Gammaproteobacteria	3831	0.0927	41	22.9
NC_021780	59454	Salmonella virus FSLSP088	dsDNA	Gammaproteobacteria	3829	0.0926	41.2	22.7
NC_031228	60606	Salmonella phage BP12C	dsDNA	Gammaproteobacteria	3797	0.0919	41.3	22.4
NC_019417	59203	Salmonella virus SPN19	dsDNA	Gammaproteobacteria	3789	0.0917	41.1	22.5
KC139515	59245	Salmonella phage FSL SP-124	dsDNA	Gammaproteobacteria	3766	0.0911	41.4	22
NC_027991	147303	Staphylococcus phage SA1	dsDNA	Firmicutes	3735.5	0.0904	41.4	21.3
NC_031930	59518	Salmonella phage 118970_sal1	dsDNA	Gammaproteobacteria	3724.5	0.0901	41.1	22.1
KC139514	59815	Salmonella phage FSL SP-039	dsDNA	Gammaproteobacteria	3696.5	0.0894	41.6	21.6
NC_021779	59746	Salmonella virus FSLSP030	dsDNA	Gammaproteobacteria	3696.5	0.0894	41.6	21.6
NC_005091	57455	Burkholderia phage BcepNazgul	dsDNA	Betaproteobacteria	3649	0.0883	42.2	21.3
NC_019524	60364	Enterobacter phage Enc34	dsDNA	Gammaproteobacteria	3619	0.0875	41.3	21.3
NC_025442	59578	Salmonella virus Chi	dsDNA	Gammaproteobacteria	3604	0.0872	42.2	20.7
NC_019411	219216	Caulobacter virus Swift	dsDNA	Alphaproteobacteria	3531.5	0.0854	36.9	19.6
NC_018832	58104	Providencia phage Redjac	dsDNA	Gammaproteobacteria	3481	0.0842	42.8	19.1
NC_029045	60216	Salmonella phage 37	dsDNA	Gammaproteobacteria	3459	0.0837	41.5	20.2
NC_019405	215710	Caulobacter virus phiCbK	dsDNA	Alphaproteobacteria	3388.5	0.082	36.4	20.6
NC_028809	58637	Pseudomonas phage PaMx74	dsDNA	Gammaproteobacteria	3373	0.0816	37.7	22.1
NC_028812	58546	Proteus phage pPM_01	dsDNA	Gammaproteobacteria	3364.5	0.0814	42.5	18.5
NC_019407	218929	Caulobacter virus Magneto	dsDNA	Alphaproteobacteria	3305.5	0.08	36	19.6
NC_021347	142563	Rhodococcus phage E3	dsDNA	Actinobacteria	3229	0.0781	36.6	19.5

ID	length	name	group	host_group	score	S_G	% mean idt.	% len
NC_018848	37612	Streptomyces phage SV1	dsDNA	Actinobacteria	3197	0.0773	35.1	23.6
NC_019410	221828	Caulobacter virus Karma	dsDNA	Alphaproteobacteria	3149	0.0762	36.3	18.8
NC_030916	56212	Tsukamurella phage TPA4	dsDNA	Actinobacteria	3147	0.0761	36	20.5
NC_007967	38194	Streptomyces phage mu1/6	dsDNA	Actinobacteria	2847.5	0.0689	36.2	20.7
NC_024145	70667	Mycobacterium phage Hosp	dsDNA	Actinobacteria	2818	0.0682	35.9	19.4
NC_023603	71565	Mycobacterium phage 39HC	dsDNA	Actinobacteria	2690	0.0651	35.9	19
NC_005885	58638	Actinoplanes phage phiAsp2	dsDNA	Actinobacteria	2621	0.0634	36	17.6
NC_028934	72139	Mycobacterium phage Vincenzo	dsDNA	Actinobacteria	2606.5	0.0631	36.7	16.5
NC_022331	69309	Mycobacterium phage Bane1	dsDNA	Actinobacteria	2605	0.063	36.8	16.3
NC_022061	70838	Mycobacterium phage KayaCho	dsDNA	Actinobacteria	2590.5	0.0627	35.4	17.6
KF279413	69306	Mycobacterium phage Bane2	dsDNA	Actinobacteria	2574.5	0.0623	36.8	16.1
NC_015210	61440	Tsukamurella phage TPA2	dsDNA	Actinobacteria	2453	0.0593	36.2	16.3
JN699004	67436	Mycobacterium phage Ares	dsDNA	Actinobacteria	2452.5	0.0593	36	15.4
NC_021319	54291	Halovirus HCTV-2	dsDNA	Euryarchaeota	2396	0.058	36	16.4
NC_011271	155372	Mycobacterium phage Cali	dsDNA	Actinobacteria	2356	0.057	36.7	14.9
NC_028869	153714	Mycobacterium phage HyRo	dsDNA	Actinobacteria	2340.5	0.0566	36.3	14.9
NC_008195	70654	Mycobacterium phage Cooper	dsDNA	Actinobacteria	2322.5	0.0562	36.2	14.9
NC_011273	164602	Mycobacterium phage Myrna	dsDNA	Actinobacteria	2293.5	0.0555	35.6	15.1
KF024722	67324	Mycobacterium virus Ta17a	dsDNA	Actinobacteria	2281.5	0.0552	35.9	14.4
NC_023600	71058	Mycobacterium phage Jolie1	dsDNA	Actinobacteria	2268.5	0.0549	36.8	15.4
NC_030913	58308	Gordonia phage Wizard	dsDNA	Actinobacteria	2270.5	0.0549	35.8	15.7
NC_008204	67188	Mycobacterium phage Qyrzula	dsDNA	Actinobacteria	2269.5	0.0549	35.8	14.8
NC_028861	44576	Mycobacterium phage FlagStaff	dsDNA	Actinobacteria	2264.5	0.0548	35.8	16
JN698991	67451	Mycobacterium phage Hedgerow	dsDNA	Actinobacteria	2257.5	0.0546	35.9	14.3
NC_004684	67480	Mycobacterium phage Rosebush	dsDNA	Actinobacteria	2249	0.0544	36.1	13.6
NC_023737	155586	Mycobacterium phage Pleione	dsDNA	Actinobacteria	2199	0.0532	36.8	13.8
NC_030848	31314	Haloarcula californiae virus 1	dsDNA	Euryarchaeota	2150	0.052	36.1	15.6
NC_011270	154906	Mycobacterium phage Spud	dsDNA	Actinobacteria	2130.5	0.0515	36.9	13.8
NC_013650	155445	Mycobacterium phage ET08	dsDNA	Actinobacteria	2122.5	0.0513	36.8	13
NC_023714	153882	Mycobacterium phage LinStu	dsDNA	Actinobacteria	2104	0.0509	35.7	14.1
NC_028931	55108	Pseudomonas phage PaMx28	dsDNA	Gammaproteobacteria	2086.5	0.0505	35.8	14.6
NC_023554	70841	Mycobacterium phage JAMaL	dsDNA	Actinobacteria	2067	0.05	36.3	14.6

Table S6. Proteins shared between AchV4 and related phages as identified by CoreGenes 5.0.

Achromobacter phage AchV4 MW269554	Achromobacter phage phiAxp-2 NC_029106	Achromobacter phage AchV4 MW269554	Bacteriophage Phobos MN478374	Achromobacter phage AchV4 MW269554	Pseudomonas phage PspYZU01 KY971609	Achromobacter phage AchV4 MW269554	Klebsiella phage YMC16/01/N133_KPN_BP MF476925
PI:QPZ53262.1 terminase small subunit	PI:YP_009226429.1 terminase small subunit	PI:QPZ53262.1 terminase small subunit	PI:QGH44987.1 terminase small subunit	PI:QPZ53262.1 terminase small subunit	PI:ASD51935.1 hypothetical protein	PI:QPZ53262.1 terminase small subunit	PI:ASW27632.1 putative terminase small subunit
PI:QPZ53240.1 terminase large subunit	PI:YP_009226430.1 DNA packaging protein	PI:QPZ53240.1 terminase large subunit	PI:QGH44988.1 terminase large subunit	PI:QPZ53240.1 terminase large subunit	PI:ASD51934.1 hypothetical protein	PI:QPZ53240.1 terminase large subunit	PI:ASW27633.1 putative terminase large subunit
PI:QPZ53301.1 head-to-tail joining protein	PI:YP_009226431.1 head-to-tail joining protein	PI:QPZ53301.1 head-to-tail joining protein	PI:QGH44989.1 head-tail adapter protein	PI:QPZ53301.1 head-to-tail joining protein	PI:ASD51933.1 hypothetical protein	PI:QPZ53301.1 head-to-tail joining protein	PI:ASW27634.1 putative head-to-tail joining protein
PI:QPZ53243.1 portal protein	PI:YP_009226432.1 portal protein	PI:QPZ53243.1 portal protein	PI:QGH44990.1 portal protein	PI:QPZ53243.1 portal protein	PI:ASD51932.1 portal protein	PI:QPZ53243.1 portal protein	PI:ASW27635.1 putative portal protein
PI:QPZ53246.1 prohead protease	PI:YP_009226433.1 36K type signal peptide peptidase SppA	PI:QPZ53246.1 prohead protease	PI:QGH44991.1 prohead protein	PI:QPZ53246.1 prohead protease	PI:ASD51931.1 prohead protease	PI:QPZ53246.1 prohead protease	PI:ASW27636.1 putative signal peptide peptidase A
PI:QPZ53252.1 major capsid protein	PI:YP_009226435.1 hypothetical protein	PI:QPZ53252.1 major capsid protein	PI:QGH44993.1 major capsid protein	PI:QPZ53252.1 major capsid protein	PI:ASD51929.1 minor capsid protein E	PI:QPZ53277.1 head decoration protein	PI:ASW27637.1 putative head decorative protein
PI:QPZ53279.1 putative tail attachment protein	PI:YP_009226438.1 hypothetical protein	PI:QPZ53279.1 putative tail attachment protein	PI:QGH44995.1 hypothetical protein	PI:QPZ53279.1 putative tail attachment protein	PI:ASD51927.1 hypothetical protein	PI:QPZ53252.1 major capsid protein	PI:ASW27638.1 putative major capsid protein
PI:QPZ53267.1 neck protein	PI:YP_009226439.1 hypothetical protein	PI:QPZ53267.1 neck protein	PI:QGH44996.1 hypothetical protein	PI:QPZ53267.1 neck protein	PI:ASD51926.1 hypothetical protein	PI:QPZ53279.1 putative tail attachment protein	PI:ASW27640.1 hypothetical protein
PI:QPZ53272.1 minor tail protein	PI:YP_009226440.1 hypothetical protein	PI:QPZ53272.1 minor tail protein	PI:QGH44997.1 hypothetical protein	PI:QPZ53272.1 minor tail protein	PI:ASD51925.1 hypothetical protein	PI:QPZ53267.1 neck protein	PI:ASW27641.1 hypothetical protein
PI:QPZ53259.1 putative major tail protein	PI:YP_009226441.1 hypothetical protein	PI:QPZ53259.1 putative major tail protein	PI:QGH44998.1 hypothetical protein	PI:QPZ53259.1 putative major tail protein	PI:ASD51924.1 hypothetical protein	PI:QPZ53272.1 minor tail protein	PI:ASW27642.1 minor tail protein
PI:QPZ53275.1 tail protein	PI:YP_009226442.1 tail protein	PI:QPZ53275.1 tail protein	PI:QGH44999.1 tail protein	PI:QPZ53275.1 tail protein	PI:ASD51923.1 tail protein	PI:QPZ53259.1 putative major tail protein	PI:ASW27643.1 major tail protein
PI:QPZ53311.1 tail protein	PI:YP_009226443.1 tail protein	PI:QPZ53237.1 tape measure protein	PI:QGH45001.1 tail length tape-measure protein	PI:QPZ53311.1 tail protein	PI:ASD51922.1 hypothetical protein	PI:QPZ53275.1 tail protein	PI:ASW27644.1 tail length tape measure protein
PI:QPZ53237.1 tape measure protein	PI:YP_009226444.1 hypothetical protein	PI:QPZ53244.1 virion structural protein	PI:QGH45003.1 capsid and scaffold protein	PI:QPZ53237.1 tape measure protein	PI:ASD51921.1 tape measure protein	PI:QPZ53311.1 tail protein	PI:ASW27645.1 hypothetical protein
PI:QPZ53244.1 virion structural protein	PI:YP_009226446.1 hypothetical protein	PI:QPZ53258.1 virion structural protein	PI:QGH45004.1 hypothetical protein	PI:QPZ53244.1 virion structural protein	PI:ASD51919.1 hypothetical protein	PI:QPZ53237.1 tape measure protein	PI:ASW27646.1 putative tail tape measure protein

PI:QPZ53258.1 virion structural protein	PI:YP_009226447.1 hypothetical protein	PI:QPZ53314.1 virion structural protein	PI:QGH45005.1 tail assembly structural protein	PI:QPZ53258.1 virion structural protein	PI:ASD51918.1 hypothetical protein	PI:QPZ53244.1 virion structural protein	PI:ASW27647.1 hypothetical protein
PI:QPZ53314.1 virion structural protein	PI:YP_009226449.1 hypothetical protein	PI:QPZ53238.1 virion structural protein	PI:QGH45007.1 hypothetical protein	PI:QPZ53314.1 virion structural protein	PI:ASD51917.1 hypothetical protein	PI:QPZ53258.1 virion structural protein	PI:ASW27648.1 putative tail assembly protein
PI:QPZ53238.1 virion structural protein	PI:YP_009226451.1 hypothetical protein	PI:QPZ53268.1 hypothetical protein	PI:QGH45020.1 hypothetical protein	PI:QPZ53238.1 virion structural protein	PI:ASD51915.1 hypothetical protein	PI:QPZ53314.1 virion structural protein	PI:ASW27649.1 putative tail assembly structural protein
PI:QPZ53261.1 putative tail fiber protein	PI:YP_009226453.1 hypothetical protein	PI:QPZ53283.1 hypothetical protein	PI:QGH44974.1 hypothetical protein	PI:QPZ53268.1 hypothetical protein	PI:ASD51900.1 hypothetical protein	PI:QPZ53238.1 virion structural protein	PI:ASW27651.1 putative virion structural protein
PI:QPZ53256.1 recombination-associated protein	PI:YP_009226481.1 hypothetical protein	PI:QPZ53295.1 transcriptional regulator	PI:QGH44978.1 hypothetical protein	PI:QPZ53283.1 hypothetical protein	PI:ASD51948.1 hypothetical protein	PI:QPZ53261.1 putative tail fiber protein	PI:ASW27652.1 putative structral protein
PI:QPZ53290.1 hypothetical protein	PI:YP_009226489.1 hypothetical protein	PI:QPZ53247.1 hypothetical protein	PI:QGH44980.1 hypothetical protein	PI:QPZ53295.1 transcriptional regulator	PI:ASD51944.1 hypothetical protein	PI:QPZ53268.1 hypothetical protein	PI:ASW27673.1 hypothetical protein
PI:QPZ53257.1 hypothetical protein	PI:YP_009226490.1 hypothetical protein	PI:QPZ53269.1 putative DNA replication protein	PI:QGH44981.1 hypothetical protein	PI:QPZ53247.1 hypothetical protein	PI:ASD51942.1 hypothetical protein	PI:QPZ53256.1 recombination-associated protein	PI:ASW27677.1 hypothetical protein
PI:QPZ53239.1 DNA primase/polymerase	PI:YP_009226419.1 hypothetical protein	PI:QPZ53296.1 hypothetical protein	PI:QGH44983.1 hypothetical protein	PI:QPZ53269.1 putative DNA replication protein	PI:ASD51941.1 hypothetical protein	PI:QPZ53295.1 transcriptional regulator	PI:ASW27688.1 hypothetical protein
PI:QPZ53247.1 hypothetical protein	PI:YP_009226423.1 hypothetical protein	PI:QPZ53245.1 DNA helicase	PI:QGH44984.1 superfamily II DNA/RNA helicase	PI:QPZ53296.1 hypothetical protein	PI:ASD51939.1 hypothetical protein	PI:QPZ53247.1 hypothetical protein	PI:ASW27620.1 hypothetical protein
PI:QPZ53241.1 DNA polymerase B	PI:YP_009226425.1 DNA polymerase			PI:QPZ53245.1 DNA helicase	PI:ASD51938.1 hypothetical protein	PI:QPZ53269.1 putative DNA replication protein	PI:ASW27621.1 hypothetical protein
PI:QPZ53296.1 hypothetical protein	PI:YP_009226426.1 hypothetical protein					PI:QPZ53296.1 hypothetical protein	PI:ASW27624.1 hypothetical protein
PI:QPZ53245.1 DNA helicase	PI:YP_009226427.1 helicase					PI:QPZ53245.1 DNA helicase	PI:ASW27625.1 putative superfamily II DNA/RNA helicase
						PI:QPZ53265.1 putative helix-turn-helix transcriptional	PI:ASW27626.1 putative HTH-domain containing protein
						PI:QPZ53302.1 putative excisionase	PI:ASW27627.1 putative excisionase

Table S7. ***Achromobacter*** **phages with completely sequenced genomes.**

	Phage name, accession number	Genome length, bp (G/C%)	Host strain	Phage family, subfamily, genus	Head size, nm tail length, nm	S_G to AchV4	Number of proteins shared with AchV4*	Number of predicted protein-coding genes, tRNAs	Reference
1.	**vB_AchrS_AchV4** MW269554	59489 (62.8%)	*Achromobacter spanius*	*Siphoviridae* --- ---	64±3.2 232±5.4			82 -	
2.	**phiAxp-2** NC_029106	62220 (60.1%)	*Achromobacter xylosoxidans*	*Siphoviridae* --- ---	~56 ~230	0.1519	26	86 -	[10]
3.	**JWF** NC_029075	81541 (60.1%)	*Achromobacter xylosoxidans*	*Siphoviridae* --- ---	~62 277 ± 17	0.0057	0	118 tRNA-Lys	[9, 14]
4.	**phiAxp-1** KP313532	45045 (56.0%)	*Achromobacter xylosoxidans* A22732	*Siphoviridae* --- ---	~66 ~230	0.0116	1 (DNAPolB)	64 -	[12]
5.	**vB_AxyS_19-32_Axy20** MK962637	46352 (56.1%)	*Achromobacter xylosoxidans* I2BC	*Siphoviridae* --- ---	58±2 118 ± 2	0.0163	1 (DNAPolB)	66 -	[13]
6.	**vB_AxyS_19-32_Axy19** MK962636	46036 (56.0%)	*Achromobacter xylosoxidans* I2BC	*Siphoviridae* --- ---	58 ± 2 118 ± 2	0.0155	1 (DNAPolB)	65 -	[13]
7.	**vB_AxyS_19-32_Axy18** MK962635	45500 (56.2%)	*Achromobacter xylosoxidans* I2BC	*Siphoviridae* --- ---	58 ± 2 118 ± 2	0.0168	1 (DNAPolB)	64 -	[13]
8.	**vB_AxyS_19-32_Axy14** MK962633	46703 (55.0%)	*Achromobacter xylosoxidans* I2BC	*Siphoviridae* --- *Steinhofvirus*	56 ± 2 145 ± 4	0	1 (DUF2800)	60 tRNA-Pro	[13]
9.	**vB_Ade_ART** MH746817	95343 (55.0%)	*Achromobacter denitrificans* PR1	*Siphoviridae* --- ---	**NA** **NA**	0.0106	2 (DUF2800, DNAPolB)	127 tRNA-Pro	-**

10.	**vB_AxyS_19-32_Axy16** MK962634	46178 (56.2%)	*Achromobacter xylosoxidans* I2BC	*Siphoviridae* --- *Steinhofvirus*	56 ± 2 145 ± 4	0.001	1 (DUF2800)	58 tRNA-Pro	[13]
11.	**vB_AxyS_19-32_Axy06** MK962627	45830 (56.3%)	*Achromobacter xylosoxidans* I2BC	*Siphoviridae* --- *Steinhofvirus*	56 ± 2 145 ± 4	0	1 (DUF2800)	55 tRNA-Pro	[13]
12.	**AMA1** MT241605	46328 (56.3%)	*Achromobacter marplatensis*	*Siphoviridae* --- *Steinhofvirus*	43 ± 0 123 ± 2	0	1 (DUF2800)	62 -	[15]
13.	**JWX** NC_028768	49714 (55.4%)	*Achromobacter xylosoxidans*	*Siphoviridae* --- *Steinhofvirus*	~62 148 ± 9	0	1 (DUF2800)	67 tRNA-Pro	[9, 14]
14.	**83-24** NC_028834	48216 (54.9%)	*Achromobacter xylosoxidans*	*Siphoviridae* --- *Steinhofvirus*	~60 126 ± 6	0	1 (DUF2800)	61 tRNA-Pro	[9, 14]
15.	**AMA2** MT241607	45901 (54.5%)	*Achromobacter marplatensis*	*Siphoviridae* --- *Steinhofvirus*	61 ± 0 120 ± 3	0	1 (DUF2800)	68 -	[15]
16.	**vB_AxyP_19-32_Axy23** MK962640	43773 (64.5%)	*Achromobacter xylosoxidans* I2BC	*Autographiviridae* --- ---	54 ± 2 -	0.0252	0	50 -	[13]
17.	**vB_AxyP_19-32_Axy09** MK962628	43287 (64.3%)	*Achromobacter xylosoxidans* I2BC	*Autographiviridae* --- ---	54 ± 2 -	0.0219	0	49 -	[13]
18.	**vB_AxyP_19-32_Axy21** MK962638	43049 (64.3%)	*Achromobacter xylosoxidans* I2BC	*Autographiviridae* --- ---	54 ± 2 -	0.011	0	49 -	[13]
19.	**vB_AxyP_19-32_Axy13** MK962632	70103 (55.8%)	*Achromobacter xylosoxidans* I2BC	*Schitoviridae* *Rothmandenesvirinae* *Inbricusvirus*	70 ± 4 n -	0	0	71 tRNA-Arg	[13]
20.	**vB_AxyP_19-32_Axy10** MK962629	73898 (54.3%)	*Achromobacter xylosoxidans* I2BC	*Schitoviridae* *Rothmandenesvirinae* *Pourcelvirus*	70 ± 4 -	0	0	83 tRNA-Phe	[13]

21.	**vB_AxyP_19-32_Axy22** MK962639	71710 (54.4%)	*Achromobacter xylosoxidans* I2BC	*Schitoviridae Rothmandenesvirinae Pourcelvirus*	70 ± 4 -	0	0	78 tRNA-Gln	[13]
22.	**vB_AxyP_19-32_Axy11** MK962630	73413 (54.3%)	*Achromobacter xylosoxidans* I2BC	*Schitoviridae Rothmandenesvirinae Pourcelvirus*	70 ± 4 -	0	0	81 tRNA-Phe	[13]
23.	**JWAlpha** NC_023556	72329 (54.4%)	*Achromobacter xylosoxidans* DSM 11852	*Schitoviridae Rothmandenesvirinae Jwalphavirus*	~59	0	0	91 -	[9, 11]
24.	**JWDelta** KF787094	73659 (54.3%)	*Achromobacter xylosoxidans* DSM 11852	*Schitoviridae Rothmandenesvirinae Jwalphavirus*	~72 /~67 (width.) 22	0	0	89 -	[9,11]
25.	**phiAxp-3** NC_028908	72825 (55.2%)	*Achromobacter xylosoxidans*	*Schitoviridae Rothmandenesvirinae Dongdastvirus*	~67 ~20	0.0009	0	80 -	[17]
26.	**vB_AxyP_19-32_Axy24** MK962641	74744 (54.9%)	*Achromobacter xylosoxidans* I2BC	*Schitoviridae Rothmandenesvirinae Dongdastvirus*	70 ± 4 -	0	0	82 tRNA-Cys	[13]
27.	**vB_AxyP_19-32_Axy12** MK962631	74096 (55.0%)	*Achromobacter xylosoxidans* I2BC	*Schitoviridae Rothmandenesvirinae Dongdastvirus*	70 ± 4 -	0	0	82 tRNA-Cys	[13]
28.	**vB_AxyP_19-32_Axy04** MK962626	73834 (54.9%)	*Achromobacter xylosoxidans* I2BC	*Schitoviridae Rothmandenesvirinae Dongdastvirus*	70 ± 4 -	0	0	80 tRNA-Cys	[13]
29.	**Mano** MT708550	42452 (64.3%)	*Achromobacter xylosoxidans*	*Myoviridae* --- ---	**NA**	0.0336	5 (TerL, portal, hypothetical HTH protein, integrase)	64 tRNA-Lys	[16]
30.	**Motura** NC_049849	221431 (53.7%)	*Achromobacter animicus*	*Myoviridae* --- *Mieseafarmvirus*	**NA**	0	0	344 tRNA-Met tRNA-Arg	-**

*****– as determined by CoreGenes 5.0; **NA** – not available; ** – no paper associated with the RefSeq/GenBank record(s).

A)

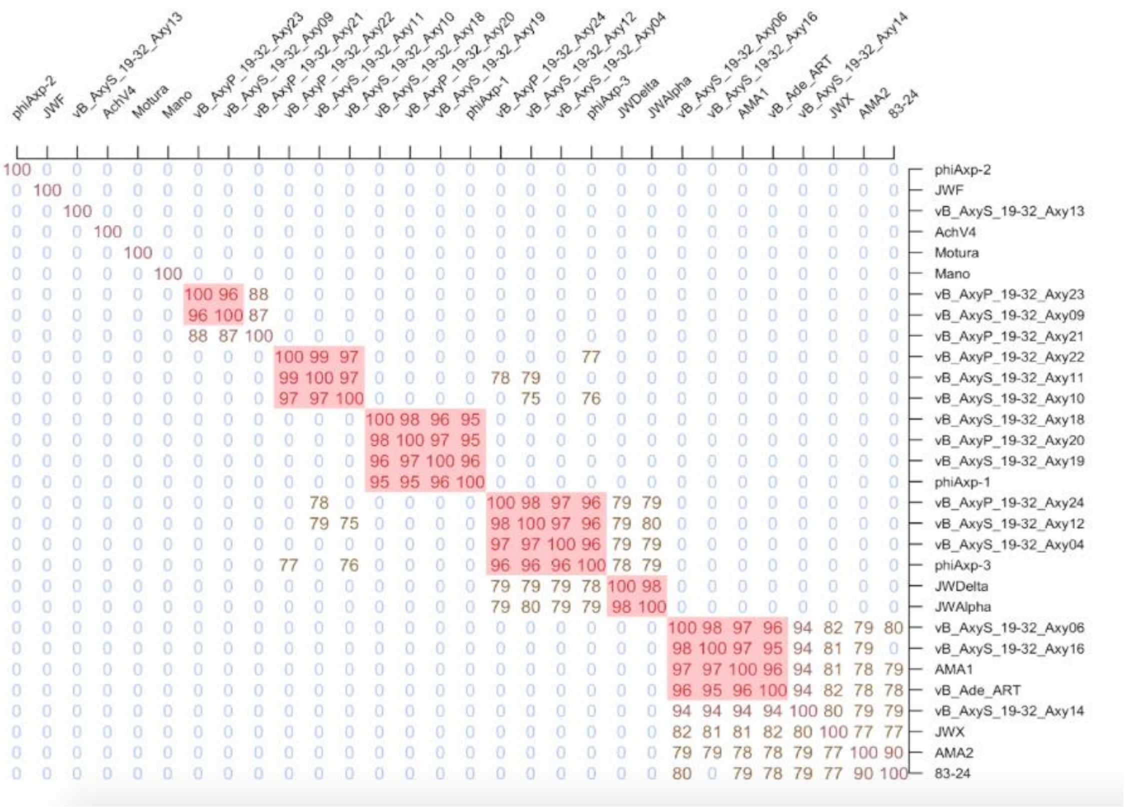

B)

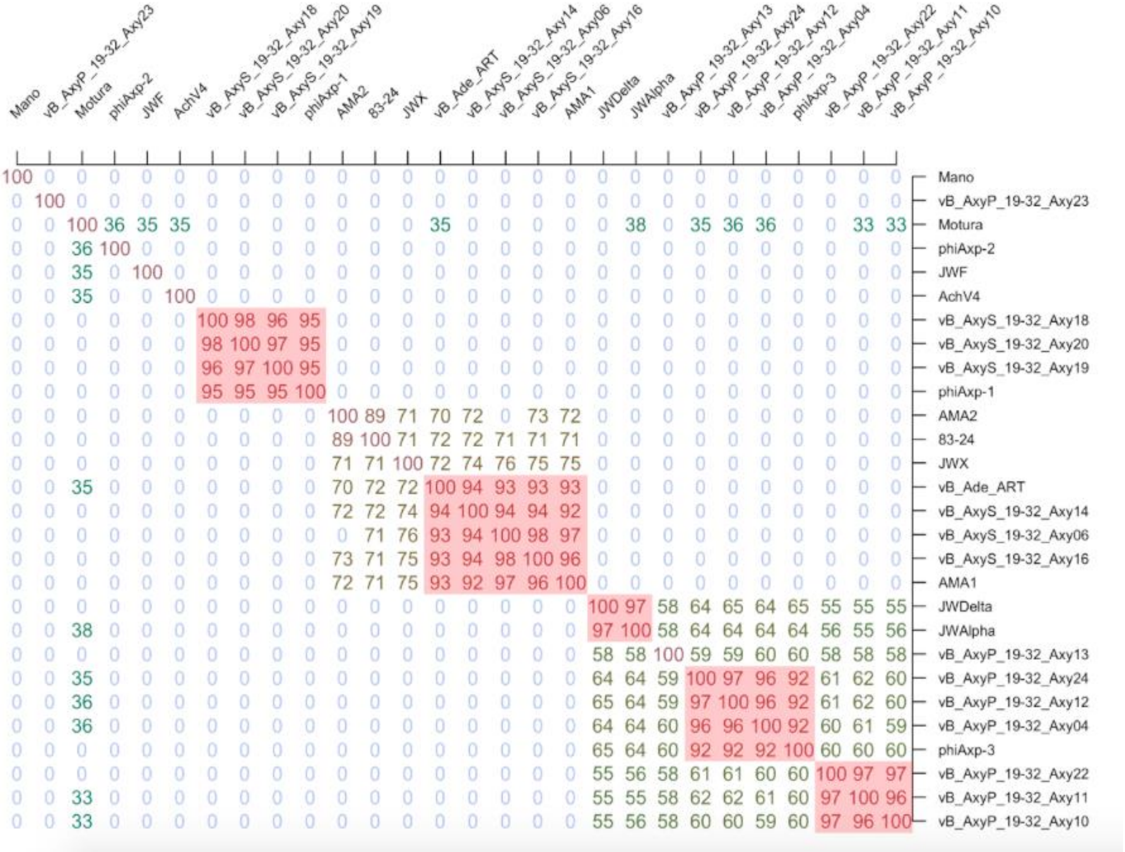

Figure S4. The average nucleotide and amino acid identities between Achromobacter phage genomes. A) average nucleotide identity (ANI) matrix; B) average amino acid identity (AAI) matrix. The figure was generated by ANI/AAI-Matrix calculator (http://enve-omics.ce.gatech.edu).

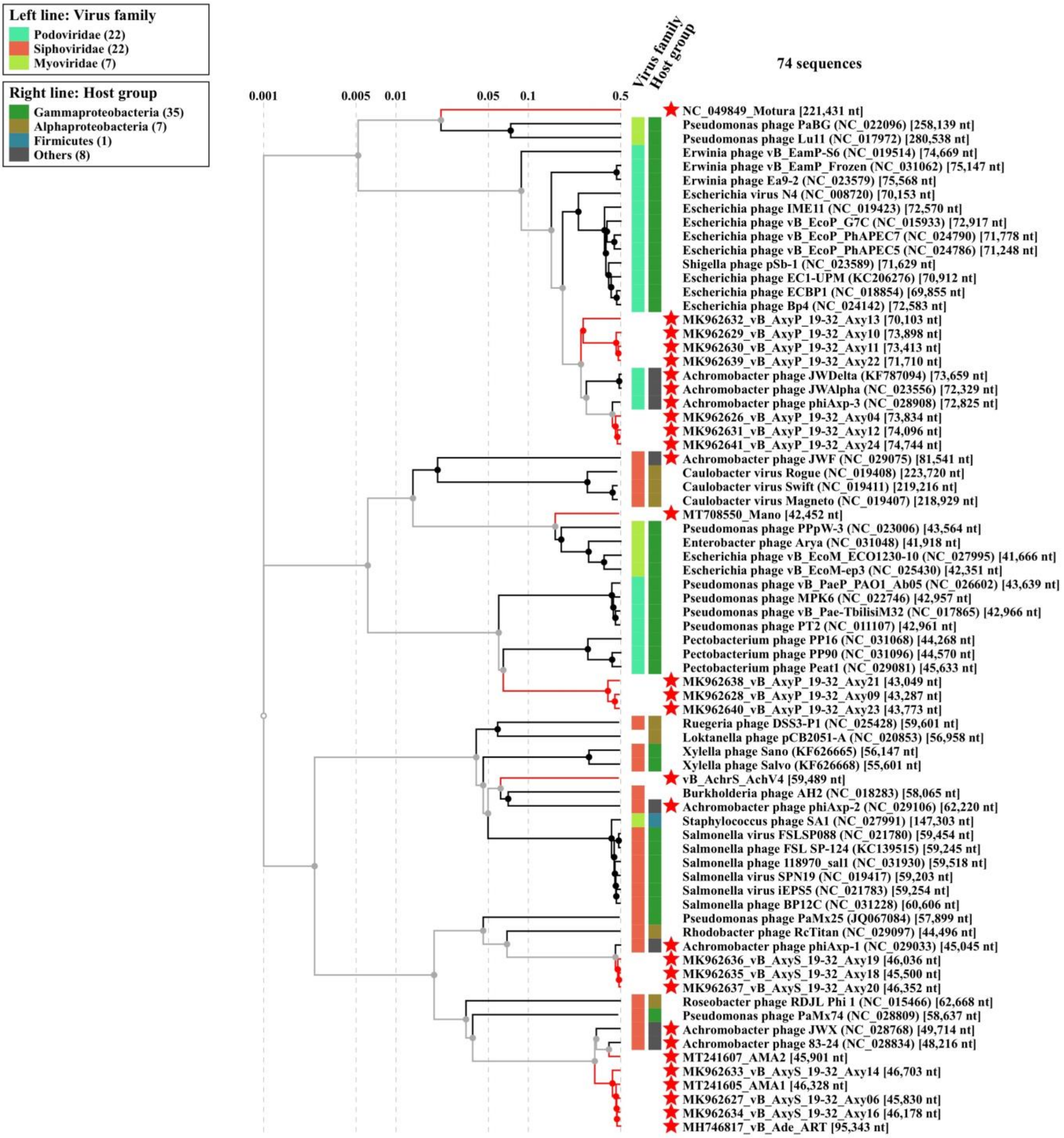

Figure S5. Proteomic tree generated based on the genome‐wide similarity relationships between 30 *Achromobacter* bacteriophages and related viruses from the Virus-Host database. The figure was generated by VipTree.

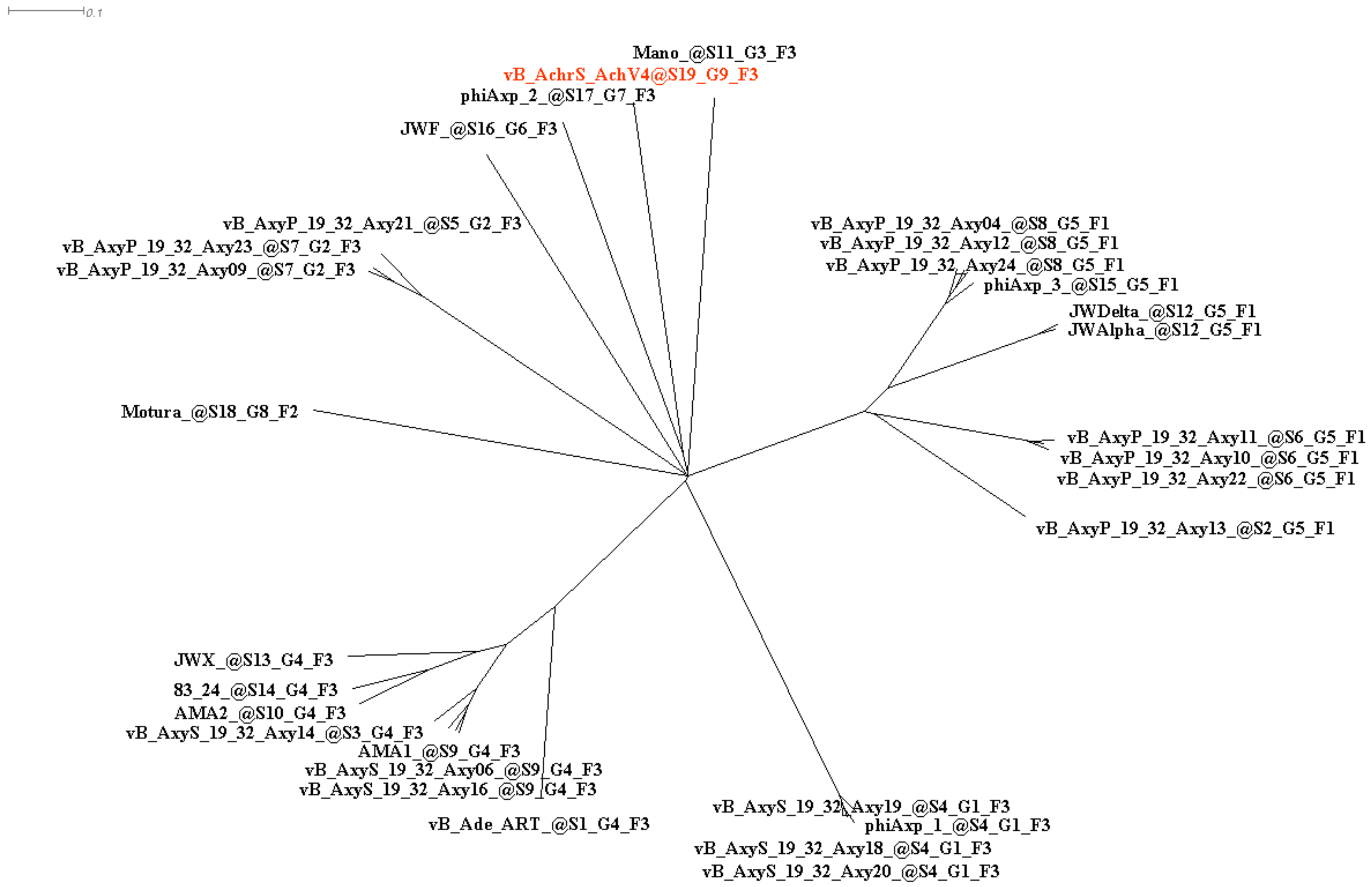

Figure S6. Splitstree representation of the unrooted phylogenomic GBDP tree obtained using VICTOR [37]. The tree inferred using the formula D6 and yielding an average support of 53 %. The OPTSIL clustering yielded nineteen species clusters. At the genus level, nine clusters resulted, respectively. The number of clusters determined at the family level were three.

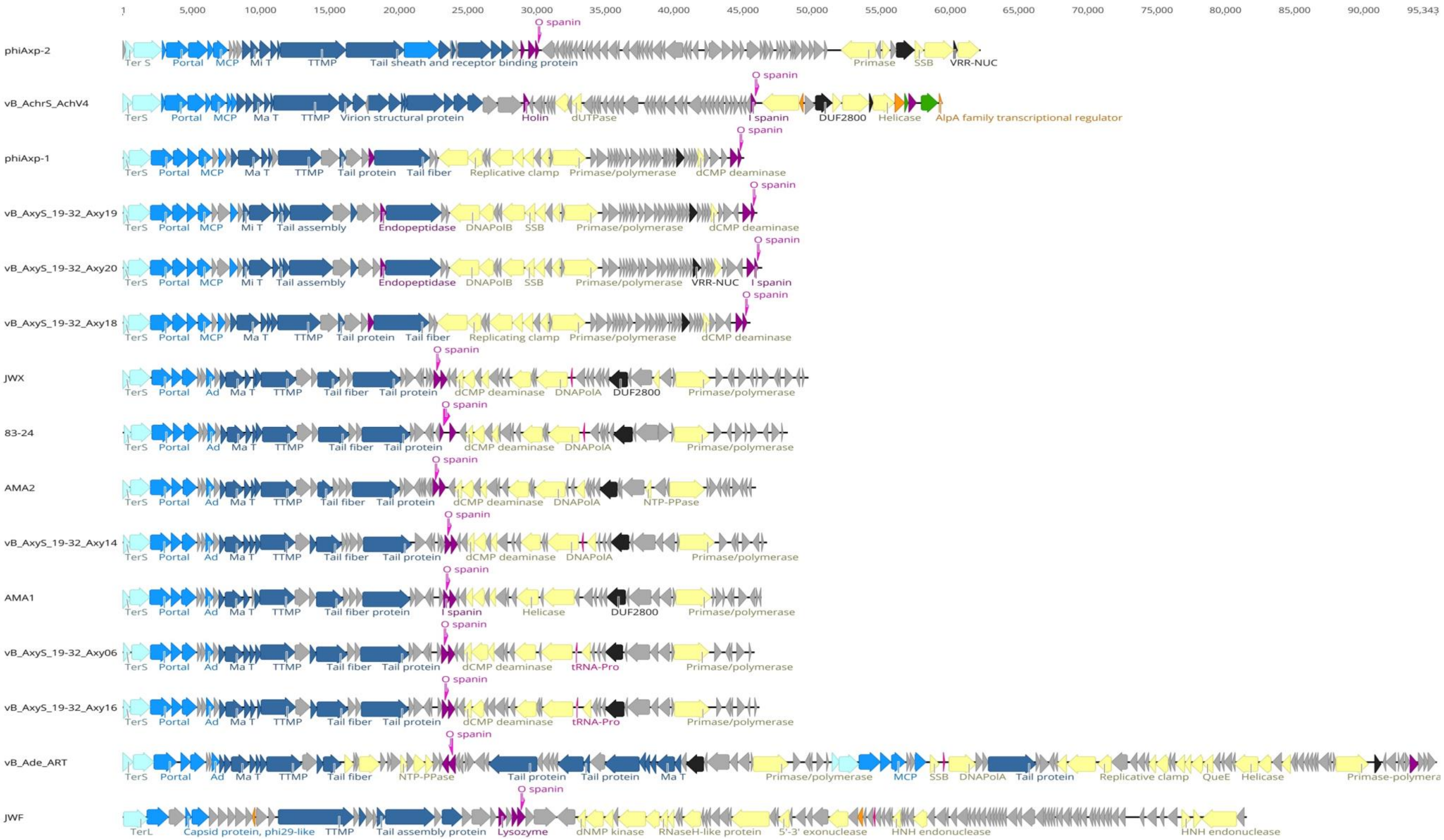

Figure S7. Schematic representation of the genome organization of *Achromobacter* siphoviruses. The colour code is as follows: light blue, terminase; blue, head cluster; dark blue, tail cluster; yellow, DNA and protein metabolism; orange, transcription; violet/purple, lysis; green, lysogeny; grey, genes of unknown function; black, conserved domain-containing proteins of unknown function. In all analyzed phages, the spanins were identified during this study. Ad – adaptor, Ma T- major tail protein, TTMP – tail tape measure protein, MCP – major capsid protein, TerL – terminase large subunit, TerS – terminase small subunit. The figure was generated using Geneious Prime v2021.01.
